# The Trinity of cGAS, TLR9, and ALRs Guardians of the Cellular Galaxy Against Host-Derived Self-DNA

**DOI:** 10.3389/fimmu.2020.624597

**Published:** 2021-02-11

**Authors:** Vijay Kumar

**Affiliations:** ^1^ Children’s Health Queensland Clinical Unit, School of Clinical Medicine, Faculty of Medicine, Mater Research, University of Queensland, St. Lucia, Brisbane, QLD, Australia; ^2^ School of Biomedical Sciences, Faculty of Medicine, University of Queensland, St. Lucia, Brisbane, QLD, Australia

**Keywords:** absent in melanoma-2-like receptors, absent in melanoma-2, Toll-like receptor 9, cyclic GMP–AMP synthase, stimulator of interferon genes, inflammation, autoimmunity

## Abstract

The immune system has evolved to protect the host from the pathogens and allergens surrounding their environment. The immune system develops in such a way to recognize self and non-self and develops self-tolerance against self-proteins, nucleic acids, and other larger molecules. However, the broken immunological self-tolerance leads to the development of autoimmune or autoinflammatory diseases. Pattern-recognition receptors (PRRs) are expressed by immunological cells on their cell membrane and in the cytosol. Different Toll-like receptors (TLRs), Nod-like receptors (NLRs) and absent in melanoma-2 (AIM-2)-like receptors (ALRs) forming inflammasomes in the cytosol, RIG (retinoic acid-inducible gene)-1-like receptors (RLRs), and C-type lectin receptors (CLRs) are some of the PRRs. The DNA-sensing receptor cyclic GMP–AMP synthase (cGAS) is another PRR present in the cytosol and the nucleus. The present review describes the role of ALRs (AIM2), TLR9, and cGAS in recognizing the host cell DNA as a potent damage/danger-associated molecular pattern (DAMP), which moves out to the cytosol from its housing organelles (nucleus and mitochondria). The introduction opens with the concept that the immune system has evolved to recognize pathogens, the idea of *horror autotoxicus*,* *and its failure due to the emergence of autoimmune diseases (ADs), and the discovery of PRRs revolutionizing immunology. The second section describes the cGAS-STING signaling pathway mediated cytosolic self-DNA recognition, its evolution, characteristics of self-DNAs activating it, and its role in different inflammatory conditions. The third section describes the role of TLR9 in recognizing self-DNA in the endolysosomes during infections depending on the self-DNA characteristics and various inflammatory diseases. The fourth section discusses about AIM2 (an ALR), which also binds cytosolic self-DNA (with 80–300 base pairs or bp) that inhibits cGAS-STING-dependent type 1 IFN generation but induces inflammation and pyroptosis during different inflammatory conditions. Hence, this trinity of PRRs has evolved to recognize self-DNA as a potential DAMP and comes into action to guard the cellular galaxy. However, their dysregulation proves dangerous to the host and leads to several inflammatory conditions, including sterile-inflammatory conditions autoinflammatory and ADs.

## Introduction

The immune system has evolved to protect the host from external pathogens and their microbe or pathogen-associated molecular patterns (MAMPs or PAMPs). The concept of *horror* *autotoxicus *introduced by the Nobel laureate Paul Ehrlich in 1899 based on experimental studies suggests that the immune system has not developed to self-attack *via* developing self-antibodies (self-Abs) or toxic Abs to endanger the host (1). However, further studies by other researchers showed the autoimmune nature of the disease called paroxysmal cold hemoglobinuria with the evidence of AutoAbs production against self-erythrocytes or red blood cells (RBCs) in 1904 (1). Also, the development of AutoAbs against self-lens protein and the eye lens-induced inflammation in patients with *endophthalmitis phacoanaphylatica* and the incidence of uveitis further strengthened the concept of autoimmunity (2). Hence, the idea of autoimmunity and autoimmune diseases evolved, and now more than 100 autoimmune diseases (ADs) are known. Therefore, the immune system may act against self-proteins and other cellular components, including genetic materials (DNAs and RNAs), once they lose their homeostatic stage at the cellular and organ level, causing a breach in the phenomenon of self-tolerance (3, 4).

The discovery of pattern recognition receptors (PRRs) called toll-like receptor 4 (TLR4) that recognizes the Gram-negative bacterial MAMP/PAMP known as lipopolysaccharide (LPS) in humans in 1997 filled the long-standing gap between the immune system and the pathogen recognition (5–7). To date, 10 TLRs (TLR1-TLR10) in humans and 12 TLRs (TLR1-TLR13) in laboratory mice have been identified, and the TLR10 in mice is a defective pseudogene (5). These TLRs recognize different PAMPs and DAMPs to elicit the NF-κB activation-dependent pro-inflammatory signaling discussed in detail by the author somewhere else (5, 8, 9). However, these TLR signaling pathways have various host-derived endogenous negative regulators, which keep their activation in check through different mechanisms (9). Hence, the TLR signaling activation pathway is a regulated pathway to protect against pathogens, PAMPs, and DAMPs, and any dysregulation causes exaggerated inflammatory signaling affecting different components of immunity causing infection-related or sterile inflammatory conditions. Hence, after TLRs, various other cytosolic PRRs, including NOD-like receptors (NLRs), absent in melanoma-2 (AIM2)-like receptors (ALRs) forming inflammasome, RIG-1 Like receptors (RLRs), cyclic guanosine monophosphate (GMP)-adenosine monophosphate (AMP) synthase (cGAS), and stimulator of interferon genes (STING) in mammals, including humans have been identified.

The inflammasomes responsible for generating mature IL-1β in response to potent inflammogen called LPS were first described in 2002 (10). Whereas ALRs (AIM2 or p210), have been discovered approximately twelve years ago in 2009 (11–14). The cGAS (a nucleotidyltransferase family member) responsible for the identification of cytosolic DNA and the induction of IRF3-dependent interferon-beta (IFN-β) or type 1 IFNs was discovered in the year 2013 (15, 16). Even the extracellular nucleosomes released due to DNA damage and apoptotic cell death taken up by cells are also recognized by cGAS as they have higher binding capacity than double-stranded DNA (dsDNA) (17). However, this cytosolic exosome recognition by cGAS does not elicit its profound activation hence low quality of pro-inflammatory cytokine and type 1 IFN generation occurs. The involvement of STING (an endoplasmic reticulum (ER) adaptor) in the cGAS signaling pathway-dependent type 1 IFN generation in response to the cytosolic DNA recognition was determined in 2008 and 2009 by the same research group (18, 19). Hence, this trinity of cytosolic PRRs recognizing cytosolic self-DNA as DAMPs is crucial to maintain cell homeostasis and harmony also in addition to recognizing pathogen-derived DNA. The present article discusses the role of the trinity of intracellular PRRs (TLR9, ALRs, and cGAS-STING signaling pathways) guarding the cellular galaxy against cytosolic self-DNAs serving as potent DAMPs to elicit several pro-inflammatory conditions, including autoinflammation, autoimmunity, and cancers.

## cGAS-STING-Based Host Cell DNA Recognition

**Figure 1:**
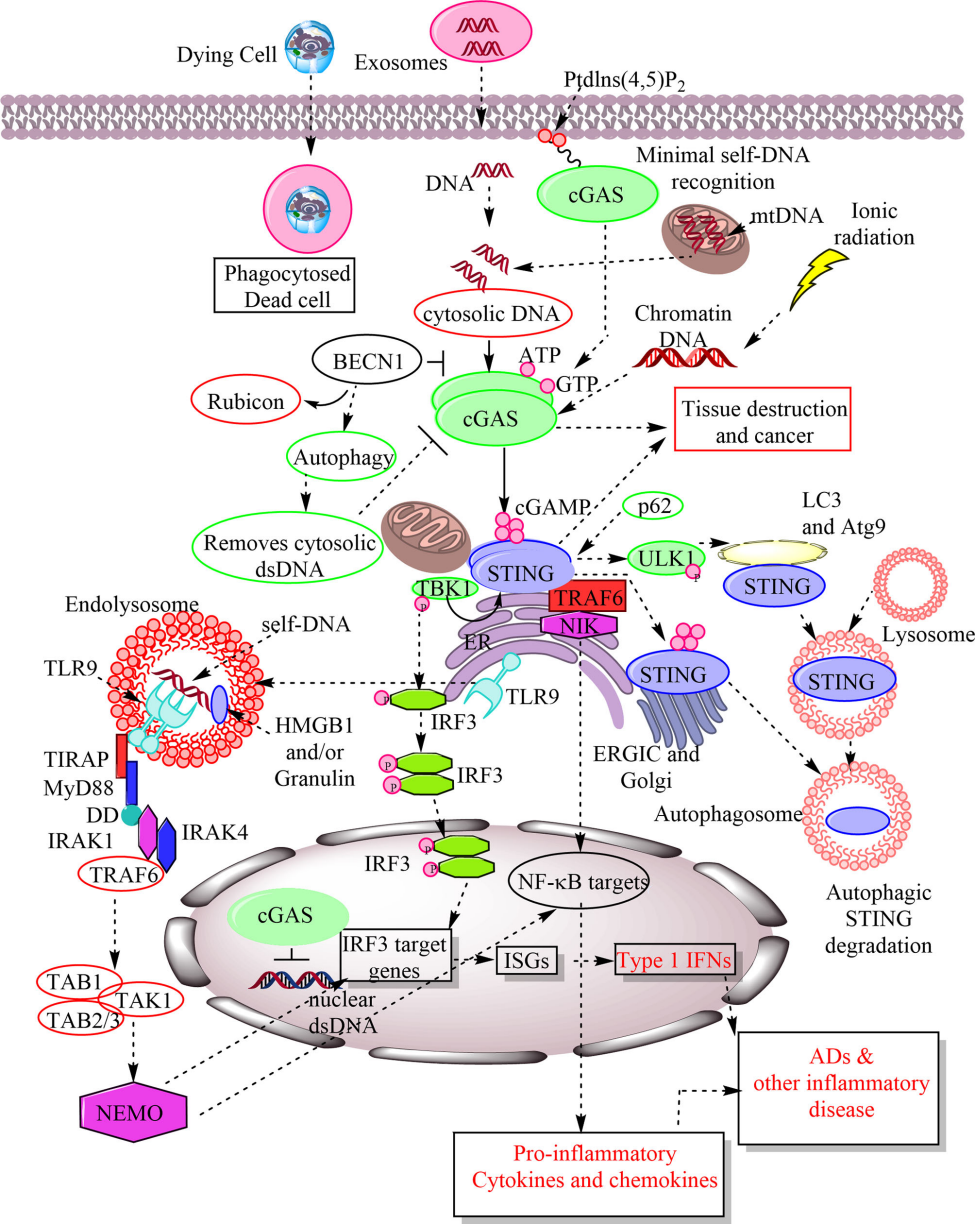
Schematic representation of cGAS-STING and TLR9 signaling in response to the recognition of host-derived self-DNA as a DAMP. The entry of the self-DNA in the cytosol due to mitochondrial damage, nuclear damage, exosome-derived DNA, and the phagocytosis of dead cell does not remain as hidden from cytosolic PRRs. The cGAS identifies them as a potent DAMP in the cytosol and catalyze the cGAMP formation. The cellular exposure to the ionic radiations also induces cytosolic levels of chromatin DNA, which is also recognized by cGAS as a DAMP. The cGAMP is recognized by downstream signaling molecule called STING located in ER. STING activation phosphorylates TBK1 that further activates or phosphorylates IRF3. IRF3 stimulates IRF3 target genes, including ISGs, which also include type 1 IFN. Also, STING activation activates NF-κB target pro-inflammatory genes for cytokines and chemokines. Hence, cGAS-STING signaling plays a crucial role in inflammation, cancer, auto-inflammation and ADs. The cGAMP recognition by STING also induces its autophagosome-mediated degradation. The p62 is an endogenous negative regulator of the STING and induces its autophagic degradation. BECN1 is also an endogenous cGAS inhibitor and their interaction removes Rubicon (an autophagy inhibitor) from the BECN1 that induces autophagy to remove cytosolic DNA. Hence, autophagy serves to remove cytosolic DNA without inducing inflammatory damage. Failure of autophagy increases inflammatory recognition of cytosolic DNAs by different cytosolic PRRs. On the other hand TLR9 is present in the ER during resting stages as soon as cytosolic CpG DNAs or host DNA enter into the endosome or endolysosomes TLR9 also migrates there and recognizes them as a crucial DAMP. TLR9 activation induces MyD88-dependent downstream signaling pathway to activate IRF3-based type 1 IFN production and NF-κB-mediated pro-inflammatory cytokine generation to cause inflammation and inflammatory diseases. MyD88 has a TIR domain and death domain (DD). The TIR domain of MyD88 activates interleukin-1 receptor-associated kinase-4 (IRAK-4) and IRAK-1. IRAK-4 subsequently recruits TRAF6 to activate transforming growth factor-β associated kinase 1 (TAK1). TAK1 is linked to TRAF6 *via* TAB2 adaptor protein, whereas TAB1 adaptor protein interacts constitutively with TAK1 and induces TAK1 kinase activity. TAK1 then phosphorylates IκB kinase (IKK) complex through K63-linked ubiquitination of NEMO (NF-κB essential modulator), an IκB kinase regulatory subunit that is critical for the NF-κB, IRF3, and MAPK signaling.

The cGAS [C6orf150 or MAB-21 domain containing protein 1 (MB21D1)]-STING signaling molecules have also evolved to serve as intracellular PRRs for the cytosolic dsDNA recognition and comprise a crucial cytosolic innate immune signaling pathway (in different innate immune cells, including fibroblasts, macrophages, and DCs) to induce type 1 IFN production in response to dsDNA viruses, retroviruses (human immunodeficiency virus-1 or HIV-1 and HIV-2), and host-derived self dsDNA (15, 20–26). The cGAS resembles the nv-A7SFB5.1 enzyme of the *Nematostella vectensi *(a sea anemone) from, which humans have evolutionarily diverged around 600 MYA (27, 28). The *N. vectensi *cGAS (nvcGAS or nv-A7SFB5.1) produces 3′,3′CDNs, which is recognized by their STING (nvSTING) through nucleobase-specific contacts absent in humans (27). Of note, nvSTING specifically recognizes the guanine nucleobases of 3′,3′CDNs. The cGAMP or cGMP-AMP (cyclic guanosine monophosphate-adenosine monophosphate) formed upon recognition of cytosolic dsDNA by cGAS (a member of nucleotidyltransferase family) binds to the STING and activates type 1 IFN production through activating interferon regulatory factor 3 (IRF3) transcription factor (TF) (**Figure 1**) (15, 29).

### Evolutionary Aspects of cGAS-STING Signaling

The researchers in 2011 first identified human cGAS (hcGAS) as an interferon-stimulated gene (ISGs) (25). The mammalian cGAMP is called 2′3′ cGAMP (contains one noncanonical 2′–5′ phosphodiester bond between G and A, and one canonical 3′–5′ phosphodiester bond between A and G) to distinguish from the bacterial cGAMP that is 3′3′ cGAMP (30–32). STING has ~10 times more affinity for 2′3′cGAMP than other cyclic dinucleotides (CDNs), and ancestral cGAS-STING pathway in *N.* *vectensis* (starlet sea anemone of the phylum cnidaria diverged from humans around 600 MYA) also binds to 2′3cGAMP more preferentially than other forms, including 3′,3′CDN produced in them (27, 33). The origin of cGAS-STING may date back to the origin of choanoflagellate (closest free-living unicellular and colonial flagellates relatives of metazoans), *Monosiga brevicollis* (34). Hence, the cGAS-STING evolution dates back to the origin of multicellularity, which is approximately 600 MYA (35). Despite, only 29% amino acid (AA) identity the crystal structure of nvSTING is identical to the human STING (hSTING) (36). Also, the STING homologs present in other invertebrates phyla, including *mollusca*, *annelida*, and *cnidarian*, have less than 30% AA identity with hSTING but bind robustly with 2′3′cGAMPs and 3′,3′CDNs (27). Hence, the CDN binding to the STING has remained conserved for more than 600 million years. However, it remains to discover the role of nvSTING in antiviral or antibacterial immune response induction except for autophagy induction that occurs independently of TANK (TRAF(TNFR-associated factor)-associated NF-κB activator)-binding kinase 1 (TBK1) activation (37).

The cGAMP binding translocates STING to the endoplasmic reticulum–Golgi intermediate compartment (ERGIC) and Golgi in a process depending on the coat protein complex-II (COP-II complex, a set of highly conserved proteins responsible for creating small membrane vesicles originating from the endoplasmic reticulum or ER) and ADP ribosylation factor (ARF) GTPases (37, 38). The heterozygous missense mutations in coatomer protein subunit alpha (COPA, a subunit of coat protein complex-I or COP-I that mediates Golgi to ER transport) cause COPA syndrome (an autosomal dominant autoimmune dysregulatory disease, involving lungs and joints) overlapping clinically with the higher type 1 IFN levels due to the gain of function in the STING, even in the absence of its ligand (39, 40). Furthermore, surfeit locus protein 4 (SURF4) serves as an adaptor molecule to facilitate the COPA-mediated STING retrieval at the Golgi-complex. Thus COPA mediates maintenance of immune homeostasis *via* regulating the STING transport to the Golgi-complex and the dysregulation of COPA overactivates STING causing immune dysregulation in the COPA syndrome (39). Another study has also shown the interaction between COPA and STING, and the mutant COPA is responsible for the accumulation of ER resident STING at the Golgi-complex (41). Hence, ER-Golgi axis also controls autoinflammation and have a potential for therapeutic approaches in the COPA syndrome. The STING with ERGIC induces LC3 lipidation (a key step in the autophagosome formation) through a pathway that depends on the WD repeat domain phosphoinositide-interacting protein 2 (WIPI2) and autophagy protein 5 (ATG5), but does not require Unc-51-like kinase (ULK) and vacuolar protein sorting 34 (VPS34)-beclin kinase complexes (37). The cGAMP-induced autophagy is crucial to clear cytosolic DNA and viruses. Thus nvSTING clears cytosolic self-DNA and viruses through autophagy without producing type 1 IFNs, indicating cGAS-STING signaling-dependent autophagy is a primordial function.

The *Drosophila melanogaster* or the common fruit fly STING called dSTING activates in response to the injected 2′3′-cGAMP and stimulates dSTING-regulated gene expression (42). The activation of immune cell deficiency (Imd) pathway in response to the viral pathogens activates the kinase dIKKβ and the transcription factor Relish, which are required for controlling the viral infection, including picorna-like viruses (43). The dSTING activation upstream of dIKKβ regulates the expression of the antiviral factor called Nazo, means enigma in Japanese (43). Hence, antiviral action of dSTING in *D. melanogaster* occurs independently of type 1 IFN production that indicates its evolutionarily conserved and ancient role in the antiviral immunity. Also, the 2′3′-cGAMP co-injection with a panel of DNA and RNA viruses in *D. melanogaster* results in the substantial decrease in the virus replication and even *D. melanogaster* lacking Atg7 and Argonaute RISC (RNA-induced silencing complex) Catalytic Component 2 (AGO2) genes (encoding autophagy and small interfering RNA pathways) also show protection against viruses upon 2′3′-cGAMP injection (42). However, *D. melanogaster* with mutations in the gene encoding the NF-κB transcription factor Relish does not show any protection against viral infections upon treatment with 2′3′-cGAMP. Also, in silkworm (*Bombyx mori*) cells cGAMP production occurs upon infection with nucleopolyhedrovirus (NPV) that is recognized by the BmSTING (44). The BmSTING deletion inhibits the antiviral immune response in the silkworm larvae due the inhibition of cleavage and nuclear translocation of BmRelish. The caspase-8-like protein (BmCasp8L) interacts with BmSTING and suppresses the BmRelish activation in the absence of cGAMP as cGAMP decreases the BmCasp8L binding to the BmSTING and increases BmRelish activation (44). The death-related ced-3/Nedd2-like caspase (BmDredd) and BmSTING interaction promotes BmRelish cleavage for efficient antiviral immune response to protect the insect cells from viral infection (44). However, upon infection with a spore forming fungus called *Nosema bombycis* the BmSTING induces microtubule-associated protein 1 light chain 3 (LC3)-mediated autophagy to protect the host (45). Hence, dSTING and BmSTING activation regulates NF-κB-mediated antiviral immune response predating the emergence of IFNs in the vertebrates. Also, the dSTING works in the mammalian cells and induces NF-κB activation (46). Of note, dSTING does not require cGAS ortholog to activate innate immune signaling pathway in *D. melanogaster*.

The *Danio rerio* or zebrafish STING (zSTING) is also capable of inducing an antiviral immune response against DNA viruses due to the presence of a conserved serine residue (S373) (47). This recognition is independent of cGAS in the zebra fish but requires zDHX9 (a Zebrafish RNA helicase) and DEAD (Asp-Glu-Ala-Asp) box polypeptide 41 (zDDX41, a member of DExD/H-box helicases superfamily that recognizes cytosolic DNA) to sense DNA viruses, including herpes simplex virus-1 (HSV-1) (47). The zDDX41 also contributes to the zSTING-zSTAT6-mediated chemokine (zCCL20) production *via* its DEADc domain (48). The zDDX41 is a trafficking protein that resides in the nucleus in resting cells and moves to the cytosol upon stimulation of cells with the cytosolic DNA. The zDDX41 serves as an initiator for the NF-κB and IFN signaling pathways activation in a zSTING-dependent manner through its DEADc domain (48). This signaling pathway protects the zebrafish from bacterial (*Aeromonas hydrophilia* or *Edwardsiella tarda*) and viral infections *via* inducing innate immunity. The C-terminal tail (CTT) of the STING has only evolved in vertebrates that is critical for TBK1 recruitment and IRF3 phosphorylation or activation (49–51). The STING CTT is an unstructured stretch of ∼40 AAs, which has sequence motifs crucial for STING phosphorylation and IRF3 recruitment (52). The human STING residue S366 serves as a primary TBK1 phosphorylation site, which is a part of the *LxIS* motif shared between innate immune adaptor proteins that activates IFN signaling (49, 50, 53). The hSTING CTT also contains a second *PxPLR* motif with a L374 residue crucial for TBK1 binding (50, 54). The *LxIS* and *PxPLR* sequences are highly conserved in all vertebrate STING alleles and serve as IRF3 and TBK1 binding sites/motifs respectively.

The zebrafish STING CTT contains a further extension that is absent in human and other mammalian STING alleles (52). The CTT of the zSTING and *Salmo salar* (Salmon) STING inverts the typical vertebrate signaling response (IRF3-dependent type 1 IFN production) and results in the dramatic (100-fold higher) NF-κB activation and weak IRF3-IFN signaling *via* recruiting TRAF6 (52). Thus, removal of CTT from zSTING prevents NF-κB activation. Hence, zebrafish CTT module is sufficient to reprogram hSTING to activate NF-κB signaling mainly along with immune activation in macrophage cells. The zSTING CTT module can mediate hyperactivation of the IRF3 reporter signaling, only in the presence of hSTING IRF3 binding module (52). This indicates that the cross-talk between individual CTT modules may affect the overall STING signaling. The STING allele from the most primitively diverged vertebrate lineage called *Callorhinchus milii* (Ghost shark) contains humans-like CTT and does not induce heightened NF-κB activation (52). Hence, the STING-dependent IRF3-IFN and NF-κB signaling depends on independent modules in the CTT, which can be gained or lost to balance downstream immune activation. Some amphibians, including *Xenopus tropicalis* (western clawed frog) and *Xenopus laevis, *have lost the CTT domain in the time of evolution (34). However, *X. tropicalis *STING can bind 2′3′cGAMP without inducing any functional response, including immune response  (27). The ability of STING to bind CDNs has remained conserved throughout metazoans and antedate the emergence of IFNs and “modern” innate immunity (55). Hence, cGAS-STING signaling pathway has evolved to protect the host *via* different mechanisms (autophagy, type 1 IFN production, and NF-κB activation) about 600 MYA and remained conserved.

### cGAS-STING Signaling in Response to the Self-DNA, its Regulation, and Impact on the Immune Response

The cGAS exists in the cells in three forms, including the cell membrane bound cGAS, freely floating in the cytosol, and in the nucleus (56). However, a study has identified that the cGAS exists predominantly as a nuclear protein independently of cell cycle phase or cGAS activation status (57). The nuclear cGAS tightly tethers to the nucleus through a salt-resistant interaction that does not require the domains crucial for the cGAS activation, but needs intact nuclear chromatin (57). The single amino acid (AA) mutation in the tethering surface of the cGAS renders it massively and constitutively active against the self-DNA. Thus the tight nuclear tethering of the cGAS maintains its resting stage and prevents autoinflammatory and autoimmune diseases. The cGAS binds to the cytosolic plasma membrane through its N-terminal phosphoinositide-binding domain that recognizes the phosphatidylinositol 4,5 biphosphate (PtdIns(4,5)P2) or PIP2, a membrane lipid (58). The mutant cGAS lacking N-terminal phosphoinositide-binding domain does not bind to the plasma membrane and moves to the cytosolic and nuclear compartments, and induces a potent immune response in response to the genotoxic stress. However, this mutant cGAS induces a weaker type 1 IFN response to viruses, including modified vaccinia Ankara (a dsDNA virus) (58, 59). The cGAS binding to the plasma membrane serves as a mechanism to prevent cGAS-binding to the cytosolic self-DNA to prevent the generation of self-destructive immune response but enhances the recognition of invading viruses. The cGAS binding to the plasma membrane varies from cell to cell, for example, non-phagocytic cells have larger cytosolic pool of cGAS than phagocytic cells (Macrophages, neutrophils, and DCs) (58, 60). This indicates the context-specific regulation of the cGAS distribution in the cellular environment.

The cGAS optimally recognizes dsDNA equal to or longer than 36 bp to initiate cGAS-STING-mediated signaling to produce type 1 IFNs and other NF-κB-dependent cytokines independent of the sequence (61–63). For example, dsDNAs with 12 bp do not activate mouse cGAS (mcGAS) efficiently (63). Similarly, dsDNAs with 16 bp can also bind to cGAS but do not induce STING activation efficiently. However, dsDNA with 18 bp can induce cGAS-STING activation in comparison to the salmon sperm DNA, a routinely used dsDNA to study the immune response in transfected cells (63). The longer dsDNAs with 20 bp have comparable activity to the salmon sperm DNA. The cGAS-dsDNA interaction involves electrostatic interactions and hydrogen (H) bond formation (63). Most of these interactions occur with bp 2 and 12 of the dsDNA. The two dsDNA binding sites (site A and site B) of cGAS are involved in these interactions, and site B is much more important than site A in this cooperative binding (63). The maximum length of the dsDNA that can stimulate cGAS activation comprises of more than 200 bp in the presence of high mobility group box protein 1 (HMGB1) and mitochondrial transcription factor A (TFAM) through inducing U-turn or curvature (64). ZCCHC3, a CCHC-type zinc-finger protein also serves as a positive regulator of cGAS *via* acting as a co-sensor and directly binding to the dsDNA that enhances cGAS binding to the dsDNA (65).

The cGAMPs or cGMP-AMPs are cyclic dinucleotides (CDNs, which were first described in bacteria), serve as second messengers during cGAS-mediated recognition of cytosolic dsDNA recognition and type 1 IFN secretion signaling pathway (16, 66). Along with activating IRF3-dependent type 1 IFN production, STING activation is also involved in the NF-κB, MAPK, and STAT6 (signal transducer and activator of transcription 6) activation, and stimulating autophagosome formation through activating LC3 puncta formation due to its co-localization with it and autophagy-related protein 9a (Atg9a, a multi-spanning membrane protein crucial for autophagy) upon recognizing cytosolic dsDNA (**Figure 1**) (49, 67–69). The deficiency or loss of Atg9a impairs innate immune response due to the enhanced assembly of STING and TBK1 (69). Hence, Atg9a also controls the STING-dependent signaling pathway in response to the cytosolic dsDNA. On the other hand, Beclin-1 (BECN1) interacts with cGAS to inhibit cGAMP formation in response to the cytosolic dsDNA *via* blocking their (cGAS and dsDNA) interaction (**Figure 1**) (70). The cGAS-BECN1 interaction releases Rubicon (a negative regulator of autophagy) from BECN1 that activates phosphoinositide 3-kinase (PI3K) class III activity to induce autophagy, which removes cytosolic dsDNA (**Figure 1**).

The phosphorylated p62/SQSTM1 inhibits the STING *via* directing the ubiquitinated STING to the autophagosome (**Figure 1**) (71). Cells deficient in p62 are unable to degrade STING, and overwhelming type 1 IFN production along with other NF-κB-dependent pro-inflammatory cytokines production takes place. Thus STING activation and p62 phosphorylation (responsible for STING degradation) occur to regulate exaggerated cGAS-STING activation in response to the cytosolic dsDNA. It will be interesting to discover factors decreasing or inhibiting p62 levels or phosphorylation in diseases associated with increased cGAS-STING-dependent type 1 IFN production. The STING activation also activates autophagy in a TBK1-independent manner that involves the translocation of the cGAMP bound STING to the ERGIC and the Golgi in a COP-II complex and ARF GTPase-dependent process (37). The ERGIC with STING acts as a source for LC3 lipidation that is a crucial step in autophagosome formation (**Figure 1**). The LC3 lipidation involves cGAMP bound to STING that comprises a WIPI2 and Atg5-dependent pathway without the involvement of ULK and VPS34 (a class III phosphoinositide 3-kinase or PI3K)-Beclin kinase complex (37).

Autophagy-related protein 16 like 1 (ATG16L1) has two distinct membrane-binding regions known as a N-terminal membrane-binding amphipathic helix involved in the LC3B lipidation and C-terminal membrane-binding region dispensable for canonical autophagy but crucial for VPS34-independent LC3B lipidation at perturbed endosome (72). The ATG16L1-C-terminus can compensate WIPI2 deletion to sustain lipidation during starvation (72). However, the C-terminal membrane-binding region is present only in the β-isomer of ATG16L1, indicating that ATG16L1 isoforms mechanistically differentiate between different LC3B lipidation mechanisms (72). The STING-mediated LC3B lipidation occurs onto single-membrane perinuclear vesicles mediated by ATG16L1 through its WD40 domain, which bypasses the requirement of canonical upstream autophagy machinery (73, 74). The WD repeat-containing C-terminal domain (WD40 CTD) of ATG16L1 is crucial for LC3 recruitment to endolysosomal membranes during non-canonical autophagy, but not for canonical autophagy (74) Bafliomycin A1 inhibits the vacuolar ATPase (V-ATPase) through binding to it. A bacterial product SpoF also inhibits V-ATPase *via* catalytically modifying it to prevent LC3B lipidation *via* ATG16L1 (73). Thus cGAS-STING signaling also induces V-ATPase-dependent LC3B lipidation to mediate cell-autonomous host defense that is different from LC3B lipidation onto double-membrane autophagosome (73).

The cGAMP-induced STING stimulation mediating autophagy, but no IFN production has been seen in *N. vectensis*, indicating that the autophagy induction through STING is primordial cGAS-STING signaling (37). However, during *Mycobacterial tuberculosis *infection, ubiquitin-mediated autophagy forms autophagosomes that degrades bacteria in response to the cGAS-mediated recognition of the bacterial DNA *via* STING-induced TBK1 activation (75). Thus the cGAS-STING signaling and autophagy induction impact each other positively and negatively may be depending on the qualities and properties of stimulating DNA or other host factors remaining to identify. The STING-dependent type 1 IFN production involves its translocation from the ER to the endosome that occurs through phosphorylation of the specific tyrosine residue (Y245) in the STING by the epidermal growth factor receptor (EGFR) (76). In the absence of STING phosphorylation through EGFR, it moves to the autophagosome where it degrades and no IRF3 activation dependent type 1 IFN production occurs as seen *in vitro* and in mice (76). Hence, EGFR tyrosine kinase regulates cGAS-STING signaling-dependent type 1 IFN production through STING phosphorylation and promoting its translocation from the ER to the endosome. Failure of this inhibits STING-dependent IFN production.

The extracellular nucleosomes ingested by cells also become a target for cGAS and have a high biding capacity for it but have lower activation potential to produce type 1 IFNs and other cGAS-STING-dependent cytokine production (17). The nucleosome recognition by cGAS may play a role in autoimmune and autoinflammatory diseases along with aggravating other inflammatory conditions. Of note, cGAS can dimerize even in the absence of dsDNA due to its intrinsic capacity to dimerize and behaves like a classic allosteric enzyme (61). Along with the cytosol, cGAS is also present in the nucleus, where chromatin tethering suppresses its activity against the self-DNA (77). The cGAS interacts with the nucleosome core particle with a nanomolar affinity through its two conserved arginine (Arg) molecules to anchor nucleosome acidic patch (comprised of histone 2A (H2A)-H2B dimer and nucleosomal DNA) that is involved in recognizing and binding to the dsDNA (78, 79). The cGAS extensively contacts with both the acidic patch of the H2A-H2B heterodimer and the nucleosomal DNA (80). Also, the cGAS engages the second nucleosome in trans. The cGAS uses two conserved arginines (Arg) to anchor nucleosome acidic patch formed by the H2A-H2B heterodimer *via* dsDNA-binding site B in both complexes (1:1 and 2;2 cGAS-dsDNA complexes), and could interact with the DNA from the other symmetrically placed nucleosome *via* the dsDNA-binding site C in the 2:2 complex (77, 81). Hence, all the three binding sites of cGAS required for self-dsDNA are not available to form the active 2:2 cGAS-dsDNA state that prevents cGAS dimerization (77, 82). The R236A or R255A mutation of the cGAS impairs its binding to the nucleosome and relives the nucleosome-mediated cGAS inhibition (81). Hence, cGAS is unable to recognize self-dsDNA inside the nucleus due to its interaction with nuclear histones (key constituents of chromatin) that prevents the onset of autoimmune or autoinflammatory diseases in response to the recognition of self-dsDNA inside the nucleus (**Figure 1**) (83). The biallelic mutations in LSM11 (U7 small nuclear RNA associated protein) and RNU7-1 (U7 small nuclear 1) encoding components of the replication-dependent histone pre-mRNA-processing complex have been detected in genetically uncharacterized cases of type I interferonopathy, Aicardi-Goutières syndrome (AGS) (83). These patients also show the altered cGAS distribution and activation in response to the chromatin lacking linker histone. However, cGAS in the nucleus interacts with the replication fork proteins in a DNA-binding manner and slows it down independent of STING to prevent the replication stress (84). Thus cGAS-deficient cells are highly sensitive to radiation and cancer therapeutics. The nuclear cGAS bound to the chromatin promotes tumor growth through suppressing homologous-recombination-mediated repair required for DNA repair (85, 86). Thus under genomic stress nuclear cGAS bound to the chromatin potentiates the genomic destabilization, micronucleus formation, and cell death independent of the STING activation (86). Hence, cGAS targeting may serve as a potential target for anticancer therapies. Thus *via* acting as a decelerator of DNA replication forks, the nuclear cGAS suppresses replication-associated DNA damage that can efficiently target to exploit genomic instability of cancer cells (84). In addition to STING-independent genomic stability, the cGAS-dependent activation of STING/TBK1/IRF3 promotes p21 or cyclin-dependent kinase inhibitor (CDKI) in the nucleus reduces micronucleus formation, delays G2/M transition, and maintains chromosomal stability (87). Hence, cGAS maintains genomic stability through both STING-dependent and independent mechanisms. Also, during mitosis-dependent cell division, the dsDNA moved in the cytosol (due to mitotic nuclear envelope break down or NEBD) escapes cGAS-dependent recognition due to its phosphorylation at S305 (in human cGAS) and S291 (in mouse cGAS) sites in response to the mitotic kinase CDK1-cyclin B complex (88). As soon as mitosis finishes, the phosphorylated cGAS gets dephosphorylated in response to the type 1 phosphatase PP1 to continue its dsDNA sensing function. Further study has shown the absence of cGAS activation along with the STING activation by the vesiculated Golgi in response to the self-DNA during mitosis and the introduction of the foreign DNA (89). However, during HIV-1 infection NONO (Non-POU Domain Containing Octamer Binding) protein binds to its capsid and activates cGAS signaling along with inducing cGAS association with the HIV DNA in the nucleus (90). Hence, nuclear cGAS can recognize viral DNA to initiate type 1 IFN production and pro-inflammatory immune response but is not available for host genomic DNA. Of note, NONO protein directly binds to the HIV-2 (weakly pathogenic) capsid with a higher affinity than the highly pathogenic HIV-1. The DNA-dependent protein kinase (DNA-PK) catalytic subunit (DNA-PKcs) encoded by the missense mutations of protein Kinase, DNA-activated, catalytic Subunit (PRKDC) are associated with autoimmune diseases due the overactivated enzymatic activity of the cGAS (91, 92). However, these patients exert an enhanced antiviral immune response. On the other hand, an acetyltranferase called KAT5 serves as a positive regulator of cGAS *via* catalysing the cGAS acetylation at several lysine residues in its N-terminal domain that promotes its DNA-binding ability (93).

Studies have shown that cGAS-STING signaling in response to the chromatin self-DNA is associated with a senescence phenotype and its (cGAS) deletion in the murine embryonic fibroblasts increases their spontaneous immortalization, and also abrogates associated senescence-associated secretory phenotype (SASP) or that is induced by DNA-damaging agents, including etoposide and radiation (94). The cytoplasmic chromatin-cGAS-STING pathway promotes SASP in primary human cells and in mice (95). The cGAS-mediated SASP production activating STING promotes senescence in a paracrine manner following irradiation and oncogene activation (96). Thus during conditions (exposure to ionic radiations) responsible for cellular senescent, the chromatin DNA in the cytosol induces cGAS-STING signaling to cause short-term inflammation for restraining activated oncogenes that promote tissue destruction and cancer (**Figure 1**) (95). The chromatin DNA recognition by cGAS-STING signaling pathway during senescence promoting conditions occurs due to the defective DNA damage response (DDR) signaling in response to the dysfunctional telomerase activity that creates a preponderance of chromatin fragments in the cytosol (97). This process occurs independently of telomerase shortening through cGAS-mediated recognition of cytosolic chromatin DNA. The cGAS-STING signaling inhibits this premature senescence and progression towards cancer. Hence, the activity of cGAS-STING signaling in both cytosol and nucleus is a highly controlled process, and any impairment may predispose the host to severe autoinflammatory or autoimmune diseases that may also develop different cancers.

The cGAS serves as a potent PRR for the recognition of cytosolic dsDNA in both plasmacytoid DCs (pDCs) and conventional DCs (cDCs), indicating their role in the generation of the potent T-cell-mediated immune response and the B cell-mediated Ab generation (20). In addition to this, STING also directly impacts adaptive immunity as its deficiency promotes the marginal zone B cell development and differentiation *via* activating B cell receptor (BCR) signaling (98). STING positively regulates SHIP-1 (SH2-containing inositol 5′polyphosphatase-1, that is required for B cell tolerance to self-antigens and dampens naïve and low-dose antigen-primed B cells) activation, but negatively regulates CD19 (a 95 kDa type 1 transmembrane protein of immunoglobulin superfamily that establishes a threshold for intrinsic B cell signaling *via* modulating BCR-dependent and independent signaling) and Bruton’s tyrosine kinase (Btk, essential for B cell development and function of mature B cells downstream to the BCR signaling) (98–101). The BCR activation in the STING^-/-^ B cells increases Wiskott-Aldrich syndrome protein (WASP, which activates downstream to the BCR signaling, links receptor signaling to the actin dynamic through actin-related proteins-2/3 (Arp2/3) complex, and also controls BCR mobility during activation) activation and F-actin accumulation *via* PI3K used by CD19-Btk axis as a central hub (98, 102). Thus, STING regulates B cell function *via* feedback from actin reorganization, indicating the positive impact of the STING on B cell function. On the other hand, STING activation in T cells induces their apoptosis, and its inhibition by Notch signaling prevents it during sepsis as T cells undergo apoptosis during sepsis (103, 104). The notch intracellular domain interacts with STING at its CDN binding site or domain that blocks the binding of the cytosolic pathogenic CDN generated by cGAS. Hence, recognition of cytosolic self-dsDNA by cGAS activates STING in CD4^+^T cells to induce their apoptosis.

The homeostatic regulation of STING involves TOLLIP (Toll-interacting protein that is an endogenous negative regulator of TLR signaling) as a stabilizer during a resting stage as its deficiency reduces STING levels in non-hematopoietic cells and tissues (105). The removal of TOLLIP from STING upon treatment with polyQ proteins *in vitro *or endogenous polyQ proteins in Huntington’s disease (HD) mouse striatum dampens cGAS-STING signaling (105). The TOLLIP deficiency in immune cells makes STING highly unstable, therefore do not produce cGAS-STING dependent type 1 IFNs in response to the cytosolic-dsDNA. On the other hand, inositol-requiring transmembrane kinase/endoribonuclease 1α (IRE1α, transduces signal of the misfolded protein accumulation in the ER called ER stress to nucleus as unfolded protein response or UPR) and lysosomes are responsible for STING degradation (105). Also, the TOLLIP deletion decreases the STING-dependent autoimmune disease in three prime repair exonuclease 1 knockout (KO) or Trex1^-/-^ mice. Thus TOLLIP serves as a STING stabilizer in resting cells that keeps a tug of war fight with its degrader IRE1α-lysosome (105). The STING signaling also plays a crucial role in sepsis, and its severity, including septic shock through different mechanisms, including increased type 1 IFN release, cell death, and impaired autophagy as autophagy has a protective role against sepsis (106, 107). The details of autophagy during sepsis have been discussed somewhere else (107). Furthermore, STING activation increases the severity of abdominal sepsis as its increased levels have been seen in circulating peripheral blood monocytes and intestinal biopsies (108). Even the STING expression in the human intestinal lamina propria of sepsis patients well correlates with the intestinal inflammation, higher circulating intestinal fatty acid-binding protein indicating enterocyte death or damage. The wild type (WT) mice subjected to the cecal-ligation and puncture (CLP)-induced sepsis show increased systemic inflammation, gut permeability, translocation of the bacteria due to the death of enterocytes in response to the recognition of the cytosolic mtDNA as STING^-/-^ mice have alleviated inflammatory immune response and decreased bacterial translocation (108). Another study has shown the protective action of STING inhibition form lethal sepsis (109). Hence, mtDNA-STING signaling inhibition may serve as a novel therapeutic approach for sepsis.

### Different Negative Regulators of cGAS-STING Signaling Pathway

Various host-derived endogenous negative regulators of cGAS-STING signaling, including post-translational modifications, have been described somewhere else (20, 59). Protein phosphatase 6 catalytic subunit (PPP6C) of protein phosphatase 6 (PP6) acts as a binding partner of Kaposi’s sarcoma-associated herpesvirus (KSHV) open reading frame 48 (ORF48) and also serves as a negative regulator of the cGAS-STING pathway (110). The PPP6C deletion enhances the dsDNA-induced and 5′ppp dsRNA-induced but not poly (I: C)-induced innate immune responses. PPP6C negatively regulates dsDNA-induced IRF3 activation through directly interacting with STING to prevent its phosphorylation but does not affect NF-κB activation (110). The PPP6C deficiency suppresses the HSV-1 and vesicular stomatitis virus (VSV) replication, and the KSHV reactivation, due to increased type I IFN production. PPP6C deficiency may also promote ADs in response to the overactivated cGAS-STING signaling-dependent cytokines and type 1 IFN production. Barrier-to-autointegration factor 1 (BAF) also serves as a natural competitor for cGAS activity for the genomic self-DNA and prevents the interaction of cGAS with the nuclear DNA by displacing the transiently bound cGAS monomers from dsDNA (111). Also, BAF limits the cGAS interaction with chromatin after nuclear envelop (NE) rupture in living cells that is consistent with the competition for DNA binding. BAF serves as a natural inhibitor of cGAS, both in the cytosol and nucleus. The loss of this negative regulation of cGAS may predispose the host to autoinflammatory or autoimmune diseases.

Immunity-related GTPase M (IRGM) also serves as a negative regulator of cGAS-STING signaling *via* interacting with cGAS to facilitate its p62-dependent autophagic degradation (112). IRGM serves as a master regulator of type 1 IFN production as IRGM^-/-^ mice and cells express higher levels of ISGs through over activated nucleic acid sensing pathways (cGAS-STING signaling and retinoic acid-inducible gene 1 (RIG-1)-mitochondrial antiviral signaling protein (MAVS) signaling pathways) and defective mitophagy causing accumulation of defunct leaky mitochondria releasing mitochondrial DNA (mtDNA) and reactive oxygen species (ROS) in the cytosol (112). Hence, IRGM maintains IFN homeostasis and protects the host from autoimmunity. Additionally, human cytomegalovirus (HCMV) tegument protein pp65 or pUL83 also inactivates cGAS without affecting STING to dampen the type 1 IFN response (113). Different mammalian viruses, including DNA virus in the *Poxviridae* family encode poxvirus immune nucleases (Poxins), which cleave 2′3′-cGAMP and serve as cGAMP nucleases to inhibit cGAS-STING signaling pathway through inhibiting STING activation (114, 115). Poxins cleave 2′3′-cGAMP through metal-independent cleavage of the 3′–5′ bond, converting 2′,3′-cGAMP into linear Gp[2′–5′]Ap[3′] (115). Furthermore, poxin homologs with 2′3′-cGAMP cleaving activity are also present in the genomes of moths and butterflies and the baculoviruses, infecting them. Hence, poxins are ancient negative regulators of cGAS-STING signaling pathway.

The Myb-like, SWIRM, and MPN domains 1 protein (MYSM1, a metalloprotease, which deubiquitinates the K119-monoubiquitinated form of the H2A) is another cGAS-STING negative regulator that upregulates after viral infection and intracellular DNA stimulation (116, 117). MYSM1 is also called 2A-deubiquitinase (2A-DUB) or KIAA1915 that is specific for monoubiquitinated H2A (uH2A) (117). MYSM1^-/-^ mice show a hyper-inflammatory immune response, acute tissue damage, and higher mortality than WT mice upon virus infection. The peripheral blood monocytes (PBMCs) isolated from SLE patients show a decreased MYSM1 expression but a higher production of type 1 IFNs. MYSM1 interacts with the STING and cleaves STING K63-linked ubiquitination to suppress cGAS-STING signaling pathway (116). In addition to the cGAS-STING signaling inhibition, MYSM1 also dampens nucleotide-binding oligomerization domain-containing protein 2 (NOD2), or caspase recruitment domain-containing protein 15 (CARD15) or inflammatory bowel disease protein 1 (IBD1)-mediated inflammation through inactivating receptor interacting protein 2 (RIP2, a proximal adaptor protein) complex that prevents the NOD2:RIP2 complex formation crucial for the inflammatory signaling pathway (118). MYSM1 selectively removes K63, K27, and M1 chains from RIP2 to prevent the NOD2:RIP2 complex formation crucial for inflammatory signaling. MYSM1 does not removes K48 polyubiquitin chians from RIP2. The MYSM1^-/-^ mice show unrestrained NOD2-mediated peritonitis, systemic inflammation, and hepatic inflammatory damage (118). Hence, MYSM1-based therapeutics may prove beneficial in cGAS-STING-based autoimmune diseases (SLE and IBD). Thus, these endogenous or different pathogen-derived molecules negatively targeting cGAS-STING signaling have a potential to target these innate immune mechanisms in different inflammatory diseases as described in following sections. However, further studies depending on the race, genetics, and sex of patients, are crucial before using cGAS-STING modulators in different cancers.

### cGAS-STING Signaling in Sterile Inflammatory Conditions or Diseases, Including Autoimmunity

The cGAS-STING signaling induced through the self-dsDNA plays a crucial role in various sterile inflammatory diseases, including ataxia-telangiectasia (AT, its patients are more prone to develop cancer), non-alcoholic steatohepatitis (NASH) or fatty liver disease (NAFLD) *via* recognizing mtDNA as a potential DAMP in Kupffer cells of the liver), the chronic exposure of STING activator (5,6-dimethylxanthenone-4-acetic acid or DMXXA) also induces NASH or NAFLD in WT mice (20, 119, 120). Also, the liver tissues of patients with NASH or NAFLD show a higher STING expression than a control group that promotes liver inflammation fibrosis (121). The increased cGAS-STING signaling is also associated with alcohol-related liver disease (ALD) *via* activating IRF3-depending type 1 IFN production (122, 123).

The cGAS-STING signaling is also involved in high-fat diet-induced obesity as deleting STING in mice protects them (124). The cGAS-STING pathway activation in adipocytes in response to the mitochondrial stress-induced mtDNA activates phosphodiesterase PDE3B/PDE4 that decreases cAMP levels and PKA signaling, which reduces thermogenesis. Also, the mtDNA damage in endothelial cells during a high-fat diet containing palmitic acid (PA) activates cGAS-STING signaling that activates IRF3, which induces intercellular adhesion molecule-1 (ICAM-1) expression inducing monocyte endothelial cell interaction/adhesion causing adipose tissue inflammation, obesity, inflammation, glucose intolerance, and insulin resistance (125). Also, PA-induced cGAS-STING activation inhibits Hippo-Yes-associated protein (YAP) activation, upregulates mammalian Sterile 20-like kinases 1 (MST1) that inhibits angiogenesis (126). Hence, cGAS-STING inhibition has the potential to decrease obesity-associated inflammation, type 2 diabetes mellitus (T2DM), NASH/NAFLD, defective wound healing, and angiogenesis. We need further studies in the field. However, obese people have an advantage over lean people in terms of their immune response against 23-valent pneumococcal vaccination due to the STING activation (127).

STING activation also plays a crucial role in bronchopulmonary dysplasia (BPD) in preterm infants (a lung inflammatory conditions induced due to hyperoxia) due to an increase in the long-non coding RNA metastasis-associated lung adenocarcinoma transcript 1 (MALAT1) that interacts with the cAMP response element-binding protein (CREB) to increase its transcription (128). MALAT1 increases HMGB1 concentration that may further activate cGAS-STING signaling by increasing the curvature of dsDNA that increases its binding potential to cGAS (129). Hence, cGAS-STING pathway inhibition may serve as a new therapeutic approach. However, further studies are required. Activation of STING increases liver perfusion injury, but in aged animals subjected to ischemia-reperfusion, it also activates NLRP3 inflammasome to further enhance the tissue inflammation *via* aggravated IL-1β and IL-18 release along with other pro-inflammatory cytokines (TNF-α and IL-6) (130). Hence, age also affects STING-mediated inflammatory diseases. However, cGAS-induced autophagy independent of STING activation protects from ischemia/reperfusion-induced liver injury (131). Hepatocytes do not express STING under normoxic conditions or after anoxia/reoxygenation. Histone deacetylase 3 (HDAC3) inhibition blocks ischemia-reperfusion-induced brain injury by inhibiting the microglial cGAS-STING signaling pathway (132). The cGAS-STING signaling activates in response to the cytosolic dsDNA, and HDAC3 promotes cGAS transcriptional expression in microglia during ischemia/reperfusion-induced brain injury. Another study has also shown that inhibiting cGAS activity with its antagonist A151 protects mice from ischemia–reperfusion–induced brain injury or experimental stroke (133). The A151 treatment decreases the cGAS expression, AIM2 inflammasome, and pyroptosis-related molecules, including caspase 1 (CASP-1), gasdermin D (GSDMD), IL-1β, and IL-18. Hence, cGAS-STING signaling also plays a crucial role in ischemia/reperfusion-induced brain injury.

STING also plays a crucial role in systemic lupus erythematosus (SLE) in response to the recognition of self-DNA *via* LYN interaction and phosphorylation to induce conventional DC (cDC) maturation and plasmacytoid DC (pDC) differentiation (134). The oxidized mtDNA released during the process of NETosis (neutrophil extracellular traps or NETs formation) also stimulates cGAS-STING signaling during SLE that further aggravates the disease (20, 135, 136). The transmembrane protein 203 (TMEM203, a conserved transmembrane protein) is an intracellular regulator of STING-mediated signaling, which interacts, cooperates, and co-migrates with STING to activate TBK1 and IRF3-dependent type 1 IFNs (137). Hence, inhibiting TMEM203 can inhibit cGAS-STING mediated cytosolic dsDNA recognition-based type 1 IFN signaling. Of note, TMEM203 is elevated in the T cells isolated from patients of SLE and is associated with the disease severity (137). The author has described the cGAS-STING signaling in response to the self-DNA somewhere else (20). Hence, STING may serve as a potential immunomodulatory target for SLE.

Mice lacking chromosome 9 open reading frame 72 (c9orf72) in their myeloid-cells show age-dependent lymphoid hypertrophy and autoinflammation as indicated by complete lack of c9orf72 gene due to the early activation of type 1 IFN signaling in DCs (138). Myeloid cells without c9orf72 show an increased STING activation upon exposure to the STING activators due to the decreased autolysosomal degradation of the STING in these cells. The STING inhibition in c9orf72^-/-^ mice prevents inflammation, splenomegaly, and increased type 1 IFN production (138). Mice lacking c9orf72 are more susceptibility to the autoimmune disease called experimental autoimmune encephalitis (EAE), an animal model for multiple sclerosis (MS). Patients with familial amyotrophic lateral sclerosis (ALS) and frontotemporal dementia (FTD) commonly have hexanucleotide repeat (GGGGCC) in the C9orf72 gene causing a decreased c9orf72 expression in the brain and peripheral blood cells (138). These patients have higher systemic type 1 IFN levels than sporadic patients of ALS and FTD due to overactive cGAS-STING signaling (in the absence of its negative regulator c9orf72) that can be reversed by the STING inhibitor.

The author has described the details of other autoinflammatory and autoimmune diseases, including type I IFN-dependent autoimmune disease, AGS, STING-associated vasculopathy with the onset of infancy (SAVI), erosive inflammatory arthritis (EIA), and psoriasis) affecting or linked with cGAS-STING signaling somewhere else (20). SAVI causes systemic inflammation characterized by vasculopathy, interstitial lung disease, ulcerative skin lesions, and premature death is an autoinflammatory disease caused by gain-of-function mutations in transmembrane protein 173 (TMEM173) gene that encodes STING (139, 140). The autosomal dominant mutations in the STING trigger IRF3 activation and subsequently upregulate ISGs in SAVI patients. However, the heterozygous STING N153S knock-in mouse model of the SAVI has shown the trigger of IRF-3-independent immune cell dysregulation and interstitial lung disease (141). Also, the mild upregulation of ISGs in STING N153S fibroblasts and splenocytes has been reported along with STING N154S SAVI patient fibroblasts. The STING N154S disrupts calcium (Ca^2+^) homeostasis in T cells and prime them to become hyper-responsive to T cell receptor (TCR) signaling-induced ER stress and UPR, causing cell death (142). This effect is mediated by the novel region of the STING called the UPR motif. The pharmacological inhibition of the ER stress prevents the cell death among STING N153S positive T cells (142). The crossing between STING N153S positive and OT-1 mice (have MHC class I-restricted, ovalbumin-specific, CD8^+^ T cells or OT-I cells) fully restores the CD8^+^T cells and drastically improves STING-induced lung disease (142). Thus STING regulates Ca^2+^ homeostasis, ER stress, and T cell survival independent of IRF3 activation or IFN production.

Another novel gain-of-function G207E STING mutation has been reported with a distinct phenotype causing alopecia, photosensitivity, thyroid dysfunction, and symptoms of SAVI (143). The treatment with the Janus kinase 1 and 2 (JAK1/2) inhibitor baricitinib seems beneficial in these patients. Overactivated STING has been also shown in MHC-matched allogenic hematopoietic stem cell transplantation (aHSCT)-induced graft-versus-host disease (GVHD) (144). This GVHD can be prevented by the early treatment with STING inhibitor soon after aHSCT. However, STING has shown protective effect during in MHC-mismatched aHSCT-induced GVHD and acute intestinal injury (145). Thus STING activation during MHC mismatched aHSCT is protective to the host but becomes harmful during MHC-matched aHSCT. Further studies are warranted in this direction. A recent study has shown the beneficial effect of Lysyl-tRNA synthetase (LysRS) in STING-dependent inflammatory diseases *via* two complementary mechanisms (146). First, LysRS interacts with RNA : DNA hybrids to delay the cGAS-mediated recognition to impede the cGAMP synthesis and secondly, the RNA : DNA hybrids activate LysRS-dependent diadenosine tetraphosphate (Ap_4_A) production, which attenuates STING-dependent signaling (146). Thus, LysRS and Ap_4_A may serve as pharmacological targets to control STING overactivation and dependent inflammatory conditions.

Trex1^D18N/D18N^ mice show an increased systemic inflammation and recapitulate many characteristics of human AGS and SLE due to the profound activation of cGAS-STING signaling and type 1 IFN production through T cells (147, 148). The cGAS deletion in Trex1^D18N/D18N^ mice prevents the systemic and multiorgan inflammation, ISG production, autoAb production and aberrant T cell activation. The Trex1 is a DNA exonuclease that regulates radiotherapy-induced tumor immunogenicity *via* degrading the cytosolic DNA generated in response to the radiotherapy (149). Also, patients with Bloom syndrome (BS, an autosomal recessive genetic disorder) lack or have a mutated BLM-RecQ-like helicase crucial for genome integrity or stability. Thus fibroblasts of BS patients have the increased accumulation of micronuclei that induces a constitutive upregulation of ISGs due to the overactivation of the cGAS-STING signaling-dependent IRF3 activation (150). BS patients also have low levels of Trex1 that increases cytosolic self-DNA and the ISG expression in BS fibroblasts. Hence, cGAS-STING signaling also plays a crucial role in the BS pathogenesis. The cGAS-STING signaling also come in action in response to the mtDNA released in the cytosol during the influenza virus infection (151). The mtDNA release in the cytosol during influenza virus infection involves viroporin activity of the influenza virus M2 protein in a MAVS-dependent manner (151, 152). However, the viral non-structural protein 1 (NS1) binds the cytosolic mtDNA and evades the cGAS activation. The cGAS expression increases in the patients with Huntington’s disease (HD, a progressive brain disorder) in response to the elevated micronuclei present in the cytoplasm of the neurons, causing their inflammatory damage and altered autophagy (153). Hence, further studies will open new avenues to target cGAS-STING signaling in these inflammatory disease.

### cGAS-STING Signaling in Cancer

During normal mitosis nucleosome (a basic repeating structural unit of eukaryotic chromatin, a single nucleosome comprises of 150–200 bp DNA wrapped around eight histone proteins) competitively inhibits cGAS activation in response to the dsDNA, and cGAS-STING signaling does not become fully functional (154). During the mitotic arrest, a low level of cGAS-STING signaling induces IRF3 phosphorylation and its accumulation that does not stimulate the type 1 IFN production but induces apoptotic cell death by alleviating Bcl-xL-dependent mitochondrial outer membrane permeabilization suppression (154). Taxol or paclitaxel (an anti-cancer agent) uses this mechanism for its anti-cancer action in mouse xenograft tumor models (154). Taxane also exerts the same effect on cGAS-STING signaling in non-small cell lung cancer (NSCLC). The cGAS-STING, TBK1, and IRF3 increasingly express in pan-cancer cells, and their gene expression level negatively correlates with their methylation in most cancer types (155). Even the high expression of cGAS-STING in some cancers decreases the inflammatory immune cell infiltration. Hence, their higher expression in some tumors well correlates with the poor prognosis. This study indicates the careful use of cGAS-STING modulators in tumor therapy, including adjuvants in tumor immunotherapies in clinics. We need further studies in the direction. The low cGAS expression in human lung adenocarcinoma patients is associated with high mortality (94). For example, STING activation in NSCLC predicts features of immunotherapy, and cisplatin treatment enhances it (156). The tumor with low STING and immune gene expression shows a high frequency of serine-threonine kinase 11 (STK11) mutation. The treatment with cisplatin increases cGAS-STING signaling and programmed death ligand-1 (PDL-1 or CD274 or B7 homolog 1) expression in different NSCLC preclinical models (156).

The activation of the STING signaling pathway in small cell lung cancer (SCLC) also enhances the protective effects of immunotherapy (157). Another study has indicated that the cGAS-STING signaling pathway inhibition due to upregulated nuclear paraspeckle assembly transcript 1 (NEAT1) in the lung cancer cells and tissues (158). NEAT1 interacts with DNA (cytosine-5)-methyltransferase 1 (DNMT1) to inhibit tumor suppressor p53 and cGAS-STING expression. The NEAT1 inhibition suppresses the lung cancer cell survival, migration, and invasion (158). NEAT1 inhibits the cytotoxic T cell infiltration in the lung cancer microenvironment to promote tumor growth in the syngeneic mouse models. Hence, cGAS-STING signaling plays a crucial role in the immune environment of different tumor microenvironments, including the lung cancer one. The STING activation has also been found beneficial in neuroblastoma *via* increasing the potent tumoricidal T cell-mediated immune response (159). The nanoparticle-based delivery of the STING activator has also increased the antitumor immune response (increased M2 to M1 macrophage polarization, IFN-γ producing T cells, tumor cell apoptosis, and CD4^+^ and CD8^+^T cell infiltration in the tumor microenvironment) in the PD-L1-insensitive triple-negative breast cancer (160). Different STING agonists are under phase I and II clinical trials (161). The results will determine their progression to the large phase III clinical trials.

## TLR9 Recognizing Self-DNA

TLRs have evolved more than 500 MYA in eumetazoan ancestors before the divergence of bilaterians and cnidarians, although they were first identified in *Drosophila melanogaster *(*D. melanogaster*) (162, 163). Of the 10 TLRs expressed in human cells, both immune and non-immune cells, only TLR9 recognizes the pathogen-derived DNA (CpG DNA) in the endolysosomes and induces the type 1 IFN production in the plasmacytoid dendritic cells (pDCs) (**Figure 1**) (164). This recognition also induces the polyclonal B cell activation in a MyD88 and interferon regulatory factor 7 (IRF7)-dependent manner (165). TLR9 localizes in the ER membrane of DCs and macrophages in their resting stage, which requires endosome shuttling to initiate pro-inflammatory signaling in response to the CpG DNA binding (**Figure 1**) (166, 167). The CpG DNA moves to the endosomes and subsequently to the tubular lysosomal compartment. Concurrent to the CpG DNA movement, TLR9 also moves from the ER to the CpG DNA containing structures, including endolysosomes, lysosomes, and endosomes (167). The TLR9 trafficking from ER to Golgi is mediated by UNC93B1 (Unc-93 Homolog B1, TLR signaling regulator) that also controls the TLR9 loading to the COPII^+^ vesicles, which originate from the ER (168, 169). These COPII^+^ vesicles deliver the TLR9 to the plasma membrane (168, 170). The UNC93B1 deficient mice show a complete loss of intracellular TLRs (TLR3, TLR7, and TLR9) in splenic DCs and macrophages (169). UNC93B1 also controls the plasma membrane localization of TLR5 that recognizes bacterial flagellin (171). Hence, UNC93B1 is crucial for TLR membrane trafficking. The strength of TLR9 signaling activation in response to the bacterial CpG DNA stimulation depends on its concentration, bacterial species (*Pseudomonas aeruginosa > Mycobacterium tuberculosis > Klebsiella pneumoniae > Escherichia coli > Staphylococcus epidermidis*), CG dinucleotide content, and the delivery of the CpG DNA inside the cell (172, 173). Further studies have indicated that the bacterial DNA binding to the TLR9 is sequence-independent and enhanced by the DNA curvature (174). The phosphodiester bond of the binding DNA induces the TLR9 dimerization independent of its sequence. Ligands with phosphorothioate (PS) backbones induce the large TLR9–DNA aggregates formation due to their propensity to self-associate. TLR9 binding site has a strong bias to bind to the phosphodiester backbone over the phosphorothioate backbone of the CpG motif (175). Thus, substituting phosphorothioate linkage for a phosphodiester linkage of just the CpG motif improves the activation potency of a phosphorothioate-based oligonucleotide for human B-cells and pDCs along with mouse bone marrow-derived dendritic cells (BMDCs) and macrophages (175).

Later studies with synthetic oligodeoxyribonucleotides (ODNs) containing CpG (unmethylated deoxycytidylyl-deoxyguanosine dinucleotide) indicate that the nucleotide sequence (nts), length, and dimerization properties of ODNs determine their propensity to bind and activate TLR9 (176). For example, ODNs with lesser than 21 nucleotides (nts), which have adenosine adjacent to their cytidine–guanosine (CG) dinucleotide motif, do not activate TLR9. The minimal ODNs activating human TLR9 comprise 2 CG dinucleotides separated by 6-10 nts, where the first CpG motif precedes through the 5′-thymidine and the poly-thymidine tail at the 3′ end of the ODN (176). However, the presence of short, CpG-containing oligodeoxyribonucleotides (sODNs) as short as two nts can enhance the TLR9 activation despite that they themselves cannot activate TLR9 (177). Hence, sODNs can enhance TLR9 activation in response to the mammalian genomic DNA even at their limiting concentration. The DNA curvature inducing proteins, including HMGB1 and histones H2A and H2C significantly enhance the TLR9 binding of the DNA (174). The cysteine rich protein granulin serving as a co-receptor for CpG DNA also coordinates their delivery to the endosomes or endolysosomes and promotes the interaction between CpG DNA and c-terminal domain of TLR9 to make ensure the TLR9 signaling activation (178, 179). Thus, cytosolic HMGB1 and granulin bind to the CpG DNA and increase their potency to bind to endosomal TLR9 and activation (**Figure 1**). Hence, TLR9 recognizes curved DNA backbones with increased curvature independent of its sequence length. Thus, increase in the curvature of the binding DNA and the presence of shorter ODNs, which themselves do not activate TLR9, further increase the binding tendency and strength of cytosolic DNA with the TLR9.

Earlier studies have indicated that the intracellular localization of TLR9 in different compartments as a strategy to discriminate between self and non-self-DNAs (180). However, in addition to the pathogen-derived CpG DNAs, TLR9 also recognizes self-DNA, including the mtDNA (which also contain CpG motif like bacterial DNA) (181–183). TLR9 has two DNA-binding sites, which functionally cooperate to promote receptor dimerization and activation (176, 184). For example, along with CpG DNA binding site, TLR9 has another DNA-binding site to bind DNA containing cytosine at the second position from the 5′ end (5′-xCx DNA). The binding of 5′-xCx DNA to the TLR9 in the presence of CpG DNA promotes TLR9 dimerization and activation. Hence, TLR9 recognizes two types of DNAs, and their binding increases its dimerization and activation. The human TLR9 (hTLR9) activation requires a pair of closely positioned CpG motifs within ODNs, but an ODN with a single CpG motif present at 4–6 nts from the 5′-end can activate murine TLR9 (mTLR9) effectively (185, 186). The ODNs, which are lesser than 23 nts and greater than 29 nts, lose their tendency to activate DCs through TLR9 activation (186). Thus, ODNs with minimal nts activate Th1 cytokine production in DCs and confirm B cell activation through increasing the expression of cell surface markers (186). Hence, the activation of TLR9 in response to the self-DNA depends on nts length and sequence. For example, due to the double CpG sequence-specificity for hTLR9, their activation decreases in response to the ODNs with a lower frequency of CpG motifs, including mammalian genomic DNA (185). This section will only describe the role of the TLR9 in recognizing self-DNAs under different circumstances or disease conditions.

### TLR9 Recognizes Self-DNA During Infections to Modify the Immune Response

Acute and chronic microbial infections, along with emerging infectious diseases (EIDs), including the present COVID-19 pandemics, always remain a threat to human life (187, 188). Although we have made advances in their immunopathogenesis and receptors, recognizing pathogens, we still need to explore the unknowns associated with infection pathogenesis. For example, some groups are resistant, and some are more susceptible to the same infection. For example, TLR9 besides, recognizing pathogen-derived CpG DNA, also recognizes host-derived self-DNA. Enterovirus 71 (EV71, which have a positive-sense single-stranded RNA (ssRNA) as a genome is a non-enveloped virus of the genus *Enterovirus *and family *Picornaviridae*) (189). EV71 (a typical neurotropic virus) is responsible for the head, foot, and mouth disease (HFMD) in children around the world that may also lead to permanent paralysis and even death due to its propensity to cause neurological disease during acute infection (190, 191). However, a recent study has indicated the comparatively increased EV71 replication in pDC isolated from TLR9^-/-^ mice than wild type (WT) mice (189). These WT DCs produce a higher amount of IFN-α, IFN-γ, TNF-α, IL-6, IL-10, and monocyte chemotactic protein 1 (MCP1) than TLR9-deficient DCs due to NF-κB activation. However, EV71 does not directly activate TLR9-dependent NF-κB activation (189). Seven days old TLR9^-/-^ mice infected with EV71 show severe neurological lesion-related symptoms (hind-limb paralysis, ataxia, and lethargy) of the disease. Hence, TLR9 activation plays a protective role in the EV71 infection, but that TLR9 activation does not involve the recognition of viral genetic material, instead uses the host-derived self-DNA that releases from cells dying due to apoptosis (189). Hence, it will be essential to explore in humans lacking TLR9 genetically and humans with single nucleotide polymorphisms (SNP) in their TLR9 allele that make it inactive and the severity of EV71 infection depending on the self-DNA recognition.

Another study has indicated that the TLR9-mediated recognition of the self-DNA during *Listeria monocytogenes *infection controls cell-mediated immunity (CMI) through a rapid conversion of conventional CD4^+^T cells to the regulatory T cells (T_regs_) (192). This process involves the CD8α^+^ DCs, which through TLR9-dependent recognition of the mtDNA (released from dead neutrophils) release IL-12p70, which generates FoxP3^+^T_regs_ from conventional CD4^+^T cells during a high dose infection, whereas a low dose infection induces CD8^+^T cell generation (192). Hence, the activation of TLR9 through self-DNA recognition determines the outcome of T cell-mediated immune response, including the generation of T_regs_, which are potential immunoregulatory T cells, and control the exaggerated inflammation. Furthermore, IL-12p70-dependent highly potent Th1-like T_regs_ inhibit allograft rejection in unmodified patients (193). Hence, TLR9 activation through recognizing self-DNA may help to lower systemic inflammation and inflammatory organ damage depending on the infection.

For example, TLR9 activation in response to the circulating mtDNA induces sepsis-induced acute kidney injury (AKI) and splenic apoptosis during polymicrobial sepsis (194). The TLR9 activation on DCs during polymicrobial sepsis promotes the IL-17A generation from γδ T cells, which induces the sepsis-induced AKI (195). The activation of TLR9 on renal tubular epithelial cells and podocytes promotes ischemic AKI through their apoptotic and necrotic cell death and inflammation as global deficiency of TLR9 does not exert any impact on murine ischemic AKI (196, 197). The activation of p38MAPK and NF-κB downstream to TLR9 signaling plays a crucial role in the podocyte apoptosis (197). Sepsis-induced AKI also involves podocyte apoptosis (198). The generation of circulating mitochondrial DNA in sepsis patients and its recognition by TLR9 also induces adaptive immune cell paralysis through suppressing the CD8^+^ T cell function to prevent organ damage (199). However, prolonged immunosuppression may predispose them to secondary infections. Thus, depending on the disease stage, activation of TLR9 in response to the self-DNA during different infections, including sepsis, may have therapeutic potential. The TLR9 inhibition during polymicrobial sepsis may protect from sepsis-induced AKI and immunosuppression (200). Senolytics also protect from TLR9 activation-mediated inflamm-aging and age-specific inflammatory responses occurring due to mtDNA recognition and increase life span (201–203). Further studies are required in the direction. Hence, TLR9-mediated self-DNA recognition exerts both protective and destructive effects depending on the pathogen, pathogen load, severity, and extent of TLR9 expression. We need further studies in the field.

### TLR9 Recognizing Host-Derived Self-DNA During Sterile Inflammatory Conditions

The chronic beryllium toxicity or exposure (both soluble and crystalline) causes the death of alveolar macrophages (AMs) that releases cellular DNA and IL-1α in the circulation (204). This also increases the CD80^hi^DCs migration in the lung draining lymph nodes (LDLNs), expressing increased TLR9 levels. The TLR9 in DCs recognize phagocytosed self-DNA and induce the expansion of pathogenic CD4^+^Th1 cells recognizing beryllium-modified HLA-DP2/peptide complex (beryllium-specific CD4^+^T cells) before the clinical development of pulmonary granulomas characterizing chronic beryllium disease (CBD) (204). Hence, the TLR9 (expressed on mobilized immunogenic DCs)-mediated recognition of self-DNA released from dying AMs plays a crucial role in CBD-induced by soluble or crystalline form. The phosphatase and tensin homolog-induced kinase 1 (PINK-1)-mediated mitophagy induces TLR9 activation in stretch-induced cell injury in response to the mtDNA that further exaggerates the inflammation in patients with mechanical ventilation (205).

#### TLR9 in Ischemia–Reperfusion Injuries

The role of TLR9 in cerebral and myocardial ischemia needs further studies as some groups have shown its activation has a protective action through activating PI3K/Akt signaling pathway during cerebral-ischemia reperfusion injury and myocardial-ischemia reperfusion injury in mice (206, 207). This protection involves an association between TLR9 and p85 subunit of PI3K, and the inhibition of PI3K/Akt activation abolishes TLR9-mediated protective action. However, a study has shown the activation of the p38MAPK signaling pathway in response to the TLR9 activation aggravates myocardial ischemia-reperfusion injury (208). Further study has shown that the inhibiting TLR9 activation with inhibitory oligodeoxynucleotide (iCpG-ODN) protects from the cerebral-ischemia reperfusion injury (209). However, HMGB1 levels rise in circulation in patients with cerebral and myocardial ischemia (210). A group has shown the protective action of cytosolic HMGB1 released from the nucleus during myocardial infarction (211). They have shown the binding of HMGB1 to the TLR9 exerts the post-myocardial repair effect through decreasing myocardial apoptosis and increasing wound healing and angiogenesis. However, the protective effect of the HMGB1 may be based on its concentration at a particular stage of cardiac injury as it exerts both protective and harmful outcomes (212). However, circulating self-DNA (both nuclear and mtDNA) serves as the marker for the severity of acute ischemic stroke or cerebral damage after acute cerebral infarction and poor outcome at three months (213, 214). The level of cell-free DNA also increases in the circulation in patients with acute myocardial infarction (AMI) (215, 216). Hence, circulating HMGB1 may increase its tendency to bind with TLR9. Further studies are required in the direction of studying the role of TLR9 in human patients of acute cerebral and myocardial infarction.

#### TLR9 Dysregulation in Early and Later in Life

The TLR9 expression dysregulation during development proves fatal to the neonatal life that depends on the type II IFN signaling driven by macrophages and IFN-γ producing NK cells (217). For example, the expression of TLR9 on transmembrane in mutant mice called TLR9^*TM*^ in their early life proved detrimental (they suffer severe or lethal hepatitis and pancreatitis, systemic inflammation, and anemia), whereas the same mutation later in life induced only mild inflammation (217, 218). The TLR9*™* bypasses the ectodomain proteolysis process before their activation and responds to the extracellular DNA, causing severe systemic inflammation and anemia without the involvement of lymphocytes (T and B cells) (218). Hence, the compartmentalization of TLR9 during embryonic development is necessary to escape from unwanted activation of TLR9 through recognizing self-DNA as a DAMP. Failure to this proves fatal to the neonate due to the ongoing development process involving apoptosis and necroptosis, causing lots of circulating self-DNA.

Both neonatal liver macrophages (Kupffer cells) and circulating Ly6hi monocytes express TLR9, but little or no TLR7 (217). Hence, TLR9*™* mutant present on cell membrane breaks the immunologic tolerance mediated by the compartmentalized location of normal TLR9 in endolysosomes, endosomes, lysosomes, and phagosomes. Endolysosomal exonucleases, phospholipase D3 (PLD3), and PLD4 (type II transmembrane proteins) degrade TLR9, and their genetic deficiency causes an enhanced TLR9 expression and TLR9-dependent severe inflammation (lethal hepatitis), causing the death of newborns within two to three weeks after birth (219). PLD4 has a narrow tissue distribution and highly expressed in DCs and myeloid cells, including macrophages and ionized calcium-binding adapter molecule 1 (Iba1)-positive microglia, but PLD3 has a broader tissue distribution than PLD4 (219, 220). The PLD3 localization to endosomes and lysosomes involves an uncommon intracellular biosynthetic route, which depends on the endosomal sorting complex required for transport or ESCRT machinery (221). The newly established anti-TLR9 monoclonal antibody (mAb) called NaR9 has a protective action against fulminant hepatitis developed in response to the over-activated TLR9 upon recognizing self-DNA and inducing systemic cytokine storm (222). Hence, NaR9 mAb has potent therapeutic properties against over-activated TLR9-mediated inflammatory diseases.

#### TLR9 in Autoimmune or Autoinflammatory Diseases

The autoantibodies (AutoAbs) to self-RNA and DNA are present in SLE patients, and TLR9 signaling engaged with B cell receptor (BCR) signaling helps in the spontaneous generation of AutoAbs against self-DNA in autoreactive B cells (223, 224). However, TLR9 does not impact the development of SLE-associated nephritis in susceptible mice (225). For example, the TLR9 deficiency aggravates the SLE due to the profound activation of lymphocytes and pDCs, and serum levels of IgGs and IFN-α increase (225). Hence, TLR9 is crucial for AutoAbs generation against self-DNA in SLE but not for inflammatory lupus nephritis. A genome-wide association study (GWAS) has also indicated that the three TLR9 polymorphisms (−1486C/T, +1174A/G, and +1635C/T) are not associated with the susceptibility to the SLE in the eastern Asian population (226). Hence, we need further studies in context of TLR9 in SLE. Also, the infants with heterozygous genotypes TLR9—1486T/C and 2848C/T show a higher frequency of cytomegalovirus infection than normal ones (227). Another GWAS has shown the association between TLR9 1174G/A polymorphism with the acute Epstein-Barr virus infection or infectious mononucleosis in children and adolescents (228). Thus, TLR9 polymorphism studies are warranted further in other autoimmune and infectious diseases along with cancers.

#### TLR9 in Obesity and Obesity-Associated Inflammatory Diseases

The circulating endogenous host-derived ssDNA and dsDNA also increase in obese people, patients with visceral obesity, and high-fat diet (HFD) fed mice (229, 230). The circulating ssDNA levels in obese people well-correlate with the homeostasis model assessment of insulin resistance (HOMA-IR) that serves as an index for insulin resistance. High circulating endogenous host-derived ssDNAs increase the pro-inflammatory M1 macrophage accumulation in adipose tissues through their recognition *via* TLR9 (230). The TLR9 expression level also increases in the adipose tissues in obese people. For example, HFD increases TLR9 expression in vascular adipose tissue (VAT), dominantly in macrophages in mice. These circulating DNAs released from degenerating adipocytes increase monocyte-chemoattractant protein-1 (MCP-1) expression in macrophages upon their recognition by TLR9. The circulating DNAs are engulfed by VAT macrophages of obese mice as indicated by their presence in the macrophage cytoplasm (230). However, macrophages from lean VAT do not increase cytosolic DNA. TLR9^-/-^ mice with HFD do not show obesity-associated inflammation, macrophage accumulation in the adipose tissue and have better insulin sensitivity than WT mice with HFD (230). Hence, TLR9-mediated recognition of the circulating self-DNA plays a crucial role in the obesity-associated inflammation and insulin resistance index.

Blocking TLR9 activity in obese people may reduce obesity-induced adipose inflammation and chances of future development of T2DM. However, the TLR9^-/-^ mice show opposite findings with the increased M1 macrophages and Th1 cells accumulation in the adipose tissue along with increased body weight and fat accumulation fed on HFD (231). Hence, we need further studies in the direction as diet, feeding type, age, sex, and genetic background of the mice may impact these results. Cytosolic HMGB1 increases in obese patients with T2DM only (232). HMGB1 in the islet of beta cells of the pancreas serves as a main stimulatory factor for insulin release. Hence, the HMGB1 bound host cytosolic or circulating DNA should be observed as HMGB1 binding to the host DNA increases its recognition by TLR9, as discussed previously.

The activation of TLR9 releasing type 1 IFNs through self-DNA, including mtDNA, also increases liver inflammation in the NASH or NAFLD through increasing hepatocytes death independent of apoptosis and liver fibrosis (233). TLR9 inhibition through ODN2088 proves beneficial in mice subjected to NASH (233). Attenuation of HMGB1 in NASH inhibits weight gain, and liver inflammation (decrease in TNF-α and MCP-1) in mice (C57BL//6) on HFD indicates that TLR9 recognizes self-DNA bound to HMGB1 (234). The development of atherosclerosis in people with obesity is another problem responsible for heart ailments along with NASH. Animal studies have shown that the angiotensin II (Ang II) infusion increases the plasma concentration of self-DNA that is recognized by the TLR9 expressed on immune cells, including macrophages, which secrete pro-inflammatory cytokines and other molecules promoting atherogenesis in the aortic arch (235). The TLR9 activation in apolipoprotein E KO (ApoE^-/-^) macrophages promotes inflammation partially through the p38 mitogen-activated protein kinase (p38MAPK) pathway activation. This circulating self-DNA in the coronary artery well correlates with the inflammatory features of coronary plaques as indicated by optical coherence tomography (OCT) in patients with acute myocardial infarction or AMI (235).

Even smoking e-cigarette (e-cig) increases the level of circulating self-DNA (mtDNA), TLR9 expression in classical macrophages, and atherosclerotic plaques (including human femoral artery atherosclerotic plaques expressing higher TLR9 levels) and lesions (236). The blockage of TLR9 before the exposure of e-cig vapours (ECVs) decreases atherosclerotic plaques or lesion formation, and the TLR9 expression increases along with lowering pro-inflammatory cytokine levels, CCR2^+^ classical blood monocytes, and the accumulation of lipids and macrophages (236). Hence, the TLR9 activation inducing a pro-inflammatory immune response in plaque macrophages in response to the circulating self-DNA is associated with vascular inflammation and atherogenesis. Circulating self-DNA binding to the TLR9 increases with its binding to the HMGB1. Studies have indicated the higher circulating levels of HMGB1 in patients with coronary artery disease (CAD) and are associated with non-calcified plaque burden with stable CAD patients (237, 238). The circulating HMGB1 levels are also associated with CAD in nondiabetic and T2DM patients (238). Thus targeting HMGB1 may indirectly suppress exaggerated TLR9 activation in these diseases. Hence, TLR9 recognizes cytosolic self-DNA under diverse inflammatory conditions and exerts protective or destruction action depending on several factors, which remain to explore.

## ALRs Recognizing Self-DNA

Different mammals have a different number of ALR genes, for example, the cow has only one ALR, humans have 4 (AIM2, IFI16 (Gamma-interferon-inducible protein Ifi-16 or interferon-inducible myeloid differentiation transcriptional activator), PYHIN1 (Pyrin and HIN (hematopoietic expression, interferon-inducible nature, and nuclear localization) domain-containing protein 1), and MNDA (myeloid cell nuclear differentiation antigen) or PYHIN3 or epididymis secretory sperm-binding protein, present only in the nuclei of cells of the granulocyte-monocyte lineage), and mice have 14 ALR genes (239–241). Of note, no PYHIN genes have been seen in non-mammalian species, along with monotremes (egg-laying predatory mammals, including duckbill platypus and echidnas) (240). The HIN domain comprises of tandem pair of oligonucleotide/oligosaccharide binding (OB) folds that are used by proteins to bind nucleic acids during replication, transcription, and translation (242). The two OB folds of HIN domain are connected by a long linker region and a conserved hydrophobic region between two OB folds holds them together tightly, forming a single compact domain (242). A single HIN domain seems to have evolved in the common ancestors of marsupials and placental mammals, which duplicated in placental mammals to give rise to three distinct forms (HIN-A, -B, and -C) (240). This indicates that they have evolved approximately not more than 200 MYA ago as the common ancestor of marsupials and placental mammals’ dates back to approximately 140 to 191 MYA (243, 244). Hence, evolutionarily ALRs are the youngest of both TLRs and cGAS PRRs. HIN-C and pyrin domains (PYD) of AIM2 have diverged from the rest of the PYHIN family, and only PYHIN protein shows orthology across different species. Hence, the defense of the genome against endogenous retrotransposons or retro-elements is an additional evolutionary driver for PYHIN proteins (240). Of note, even within the same gene, the pyrin and HIN domain phylogeny is not compatible, indicating that the recombination may have led not only to species-specific expansions of ALR genes but also scrambled the existing genes into novel combinations of Pyrin and HIN domains (239). This indicates that ALR genes in mammals exhibit remarkable plasticity, and no single ALR gene is preserved among all mammals with a little preserved orthology across species. ALR genes have undergone extensive species-specific diversification that indicates the presence of great evolutionary pressure that has shaped the ALR sequences and function throughout whole mammalian lineages (239).

**Figure 2:**
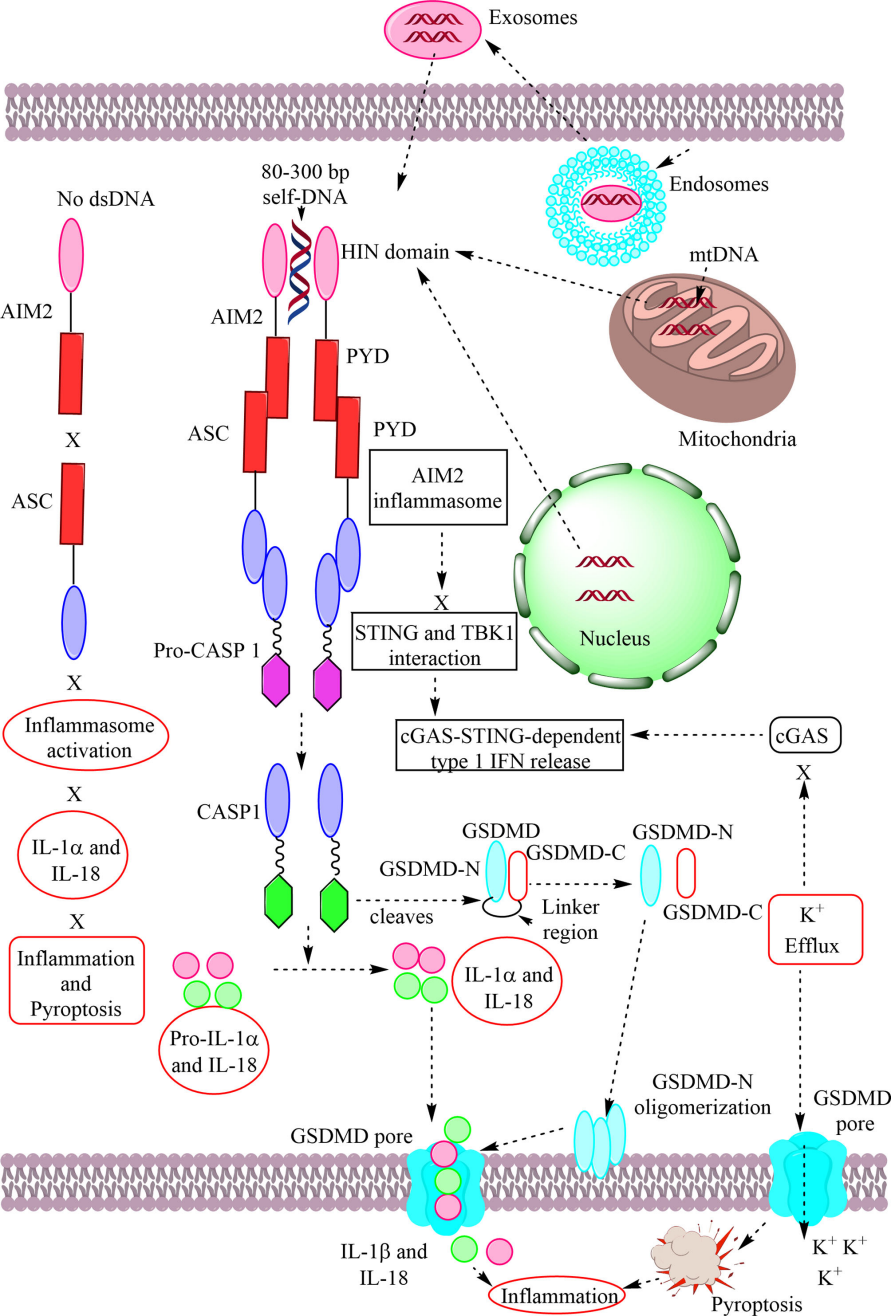
Schematic representation of AIM2 in response to self-DNA. AIM2 is an ALR that becomes activated upon recognizing and binding to the self-DNA coming into cytosol due to cellular damage, including mitochondrial and nuclear damage, and exosome with host DNA. AIM2 activates efficiently in response to self-DNA with 80–300 bp. The HIN domain of the AIM2 recognizes cytosolic DNA and its PYD interacts with the PYD of the ASC to form inflammasome complex that activates procaspase 1 (pro-CASP1) into CASP1. The CASP 1 cleaves pro-IL-1α and -IL-18 into IL-1α and IL-18. CASP1 also cleaves the linker region of the GSDMD and frees GSDMD domains (GSDMD-N and GSDMD-C). The free GSDMD-N terminals interacts with phosphoinositides or other acidic lipids to oligomerize and form GSDMD pore. The GSDMD pore mediates the IL-1α and IL-18 release from the cells. Also, the K^+^ efflux from the GSDMD pore inhibits cGAS activity and the cGAS-STING mediated type 1 IFN release along with inducing pyroptosis. The AIM2-induced GSDMD acts as a negative regulator of cGAS-STING-mediated type 1 IFN production. Also, AIM2-ASC inflammasome inhibits STING-TBK1 interaction required for IRF3-dependent type 1 IFN release. The AIM2 remains inactive in the absence of specific cytosolic DNAs.

For instance, the two factors for the dramatic differences in the number and sequences of mouse and rat ALRs include gene expansions in the mouse of three ancestral rodents ALRs, and independent reassortment of the Pyrin and HIN domains, which create an extra diversity. The three human ALRs (aside from AIM2) are not represented within these three rodent-specific clusters. All murine ALRs relocalize when expressed with relevant adaptor proteins (STING and ASC), and co-expression of an ALR with a single adaptor molecule reveals indiscriminate co-localization (239). Murine ALRs show three predominant patterns of localization: (1) AIM2, MNDA, and MNDAL co-localize with ASC adaptor proteins of inflammasome mainly, with a minimal co-localization with STING, (2) PYHIN-B, PYR-A, PYHIN-1, PYHIN-A, IFI204, IFI203, and IFI205 mainly co-localize with STING in a structure called STING-positive-ER-Golgi complex, together with concomitant recruitment of ASC to these areas of ALR-STING co-localization, and (3) The rest three ALRs (PYBLHIN-C, PYR-RV1, and IFI202B) colocalize with the puncta of ASC and not with STING (19, 239). Hence, murine ALRs can recruit ASC, STING, or both depending on their co-localization. AIM2 robustly activates ASC inflammasome to release IL-1β and IL-18 (**Figure 2**).

AIM2 or p210 belongs to the family of p200 or HIN-200 proteins (hematopoietic interferon-inducible nuclear proteins with a 200 amino acid (AA) repeat) or PYHIN (IFI200/HIN-200) protein family (pyrin and HIN-200 domain-containing proteins, which have a DNA-recognizing innate receptors family, including ALRs (245, 246). AIM2 has one HIN domain that binds to the cytosolic dsDNA and one PYD (**Figure 2**) (246). The binding of the HIN domain (contains two oligonucleotide/oligosaccharide binding (OB) folds with great affinity to DNA) to the cytosolic dsDNA promotes the ASC [an adaptor molecule, an apoptosis-associated speck-like protein containing a CARD (Caspase activation and recruitment domain)]-dependent inflammasome assembly through pyrin-pyrin domain interaction to produce mature IL-1β and IL-18 (**Figure 2**) (14, 247–249). AIM2 can be activated by mtDNA, nuclear DNA released in the cytosol due to nuclear death, and self-DNA secreted by exosomes (**Figure 2**) (250, 251). Exosomes are extracellular vesicles (EVs) with a diameter ranging from 40–160 nm (average ~100 nm) and have an endosomal origin (**Figure 2**) (252). Under homeostasis, AIM2 exists as in an autoinhibitory stage due to an interaction between HIN and PYD domain that blocks the availability of PYD for ASC PYD (**Figure 2**) (253).

The binding of cytosolic dsDNA to the HIN domain of the AIM2 does not depend on the DNA sequence and its GC sequence but depends on its length that should have at least 80 bp (13, 254, 255). A dsDNA of ~80 bp may accommodate a maximum of 20 HIN domains of AIM2 (255). However, a dsDNA with ~200 bp allows an optimal AIM2 activation. The dsDNA with ~300 bp induces a significant AIM2 polymerization into filaments than dsDNA with ~72 bp (255, 256). Hence, cytosolic dsDNA binding to the AIM2 depends on its length that further determines its kinetics and magnitude of AIM2 inflammasome activation. Of note, the AIM2 PYD suppresses the HIN: DNA interaction, despite lacking a DNA-binding capacity. Thus, dsDNA binding to the AIM2 HIN domain displaces the PYD from its intermolecular complex to facilitate the PYD downstream signaling to the ASC protein (255). AIM2 HIN domains (consist of two oligonucleotide/oligosaccharide binding (OB, OB1, and OB2) fold and a linker between them) bind both grooves of the dsDNA involving both minor and major grooves indicating their specificity for dsDNA binding or recognition only (255). The DNA interface from the AIM2 OB1 is centered at residues K162 and K163 between β1 and β2’ strands and K198 and K204 near the α1 helix. The OB1-OB2 linker contains amphipathic α2-α3 helices, which form hydrogen (H)-bonds and van der Waals (vDW) contacts from R244, K251, or G247, and T249 for different AIM2 HIN domains (255). The OB2 of AIM2 HIN forms salt bridges and vDW contacts with DNA through residues R311 at the β4 strand and residues K335 and I337 at the β5 strand. R311 faces the minor groove of the dsDNA faces R311 and forms bidentate H-bonds with the phosphate backbone of DNA.

AIM2 does not have an oligomerization domain, and electrostatic interaction between the HIN domain and cytosolic dsDNA takes place to activate AIM2 (255). This dsDNA-HIN domain interaction of AIM2 releases the signaling PYD from its intermolecular complex containing the HIN domain. It defines the multivalent ligand dsDNA as the oligomerization platform for forming inflammasome/pyroptosome complex (255) The AIM2 binds to the ASC only after the release of auto-inhibition *via* binding to the cytosolic dsDNA (**Figure 2**) (257). Hence, AIM2, without its bound ligand, is unable to activate ASC-dependent inflammasome and pyroptosome formation to induce pro-inflammatory cytokine release and pyroptosis (**Figure 2**). The PYD of the AIM2 drives the filament formation and dsDNA binding (258). The HIN domain of the AIM2 that comprises the dsDNA binding domain also oligomerizes and assists in the filament formation. Hence, the ability to oligomerize is critical for dsDNA binding that permits the size of the dsDNA to regulate the AIM2 polymers assembly. The AIM2 pyrin oligomers define the filamentous structure (258). The helical symmetry of the AIM2 pyrin filament is consistent with the filament assembled by the PYD of the downstream adaptor ASC. Hence, the AIM2 PYD is not auto-inhibitory, but the generation of the structural template by coupling ligand binding (dsDNA) and oligomerization serves as a crucial signal transduction mechanism in AIM2 inflammasome (258). Thus AIM2 oligomerizes on cytosolic dsDNA that initiates the nucleation of ASC adaptor filament, inducing the pro-caspase-1 (pro-CASP1) filament polymerization activating caspase 1 (CASP1) through auto-proteolysis to produce pro-inflammatory cytokines (IL-1β and IL-18) and induce pyroptosis (**Figure 2**) (12–14, 249, 258). Hence, minimal oligomer assembly requires six protomers, and the optimal one needs ~24 protomers (258).

ALR activation is not essential for inducing type 1 IFN production in response to the cytosolic DNA and also does not contribute to the autoimmune disease in the Trex1^-/-^ mice with AGS (259, 260). Another study has indicated that mouse ALR IFI205 senses cytosolic retrotransposon DNA independently of cGAMP production, and this process does not produce type 1 IFNs as this process prevents its recognition by STING (261). ASC is also called PYCARD (PYD and CARD containing protein)/Target of Methylation-induced Silencing-1 (TMS1) and serves as a central adaptor molecule in the inflammasome complex-dependent pro-inflammatory cytokine release (248, 262). Also, to release mature pro-inflammatory cytokines (IL-1β and IL-18), the interaction between ASC and AIM2 forms ASC pyroptosome that induces pyroptosis in cells containing CASP1, including macrophages (**Figure 2**) (12). The AIM2 inhibition prevents cytosolic DNA recognition and the inflammasome/pyroptosome activation in macrophages. Hence, recognition of the cytosolic DNA by AIM2 induces their oligomerization and AIM2 inflammasome formation to release pro-inflammatory cytokines and induce pyroptosis as an indicator for the cell’s internal danger.

Another, dsDNA-binding protein called p202 inhibits the AIM2 signaling in some mouse strains (11). For example, New Zealand Black (NZB) mice express p202 (an endogenous inhibitor of AIM2 activation) and therefore do not secrete a high amount of IL-1β and IL-18 upon stimulation with dsDNA (263). NZB mice develop anti-erythrocyte Abs and serve as an animal model for autoimmune hemolytic anemia. However, NZB mice also lack another inflammasome protein called the NLR family, PYD containing 3 (NLRP3) due to the point mutation in the NLRP3 gene. The HIN1 domain of the mouse p202 binds to the dsDNA on the opposite site used in AIM2 whereas HIN2 forms a homotetramer that increases its avidity for the dsDNA (264). However, HIN2 of p202 also interacts with the HIN1 of the AIM2 resulting in the spatial separation of the AIM2 PYDs, causing p202-mediated prevention of the dsDNA-dependent clustering of ASC and AIM2 inflammasome activation (264). The 25-hydroxycholesterol (25-HC) production through the activation of cholesterol-25-hydroxylase (Ch25h) that maintains the repression of sterol regulatory element-binding protein 2 (SREBP2, a master regulator of sterol and fatty acid synthesis) activation by macrophages also prevents AIM2 inflammasome activation (250, 265).

Some viral proteins, including HSV-1 tegument protein VP22 and human cytomegalovirus (HCMV) tegument protein pp65 (pUL83) inhibit AIM2 activation *via* direct interaction preventing its oligomerization (an initial step in the AIM2 inflammasome activation) (266, 267). Also, the HCMV immediate early 86-kDa protein (IE86) inhibits the AIM2-mediated release of mature IL-1β *via* associating with the block in the transcription of the pro-IL-1β gene (268). Hepatitis B virus (HBV) hepatitis B e antigen (HBeAg) suppresses the AIM2 inflammasome activation in the chronic hepatitis B (CHB) patients that may induce HBV-induced immunotolerance (269). The post-translational modification plays decisive role in the activation of inflammasome, including AIM2 (270, 271). For example, tripartite motif 11 (TRIM11) inhibits AIM2 inflammasome activation *via* binding to it through its PS domain and undergoes auto-polyubiquitination at K458 to promote TRIM11 and the autophagic cargo receptor p62 association to mediate AIM2 degradation *via* selective autophagy (272). Hence, these proteins or their synthetic homologs have a potential to target AIM2-meditated inflammatory diseases. Additionally, vitamin B_2_ or riboflavin also inhibits AIM2 inflammasome activation through preventing the mitochondrial damage and the release of ROS and mtDNA in the cytosol (273).

The AIM2 activation serves as an endogenous negative regulator of cGAS-STING signaling and the type 1 IFN production through GSDMD that depletes intracellular potassium (K^+^) *via* forming membrane pores inducing pyroptosis (**Figure 2**) (20, 274–277). GSDMD is a 480-AA protein that contains two defined domains [GSDMD-C (22 kDa) and GSDMD-N (31 kDA)] linked by the linker region (**Figure 2**) (278). The association of GSDMD-N with GSDMD-C through the linker region inhibits the pyroptosis induction (279). The inflammasome-mediated CASP1 activation cleaves the GSDMD linker region and forms a non-covalent complex between the N terminus (GSDMD-N) and C terminus (GSDMD-C) (**Figure 2**). The cleaved N terminus or GSDMD-N auto-oligomerizes on membranes upon encountering phosphoinositides or other acidic lipids to form large circular pores called GSDMD pore (278). The GSDMD pores are essential for IL-1β release from living macrophages exposed to the inflammasome activators, including bacteria and their PAMPs/MAMPs or host-derived oxidized lipids (280). GSDMD pores are required for IL-1β transport across an intact lipid bilayer. Hence, a non-pyroptotic function of the indicates the possibility of GSDMD pores serving as conduits for the secretion of cytosolic cytokines under the condition responsible for cellular hyperactivation (280).

Disrupting the interaction between GSDMD-N and phosphoinositides or other acidic lipids or GSDMD-N oligomerization suppresses the cell killing or death through pyroptosis due to inhibition of GSDMD pore formation (281). Hence, inflammasome activating conditions determine the pyroptosis-mediated cell death through the GSDMD activation or the release of pro-inflammatory cytokines (IL-1β and IL-18) form the living hyperactive cells with intact plasma membrane through GSDMD pore. For example, oxidized phosphorylcholine-derivatives called oxPAPC (produced from dying cells at site of tissue injury and are considered LPS-like DAMPs) are recognized and captured by CD14 expressed on macrophages and DCs (282). CD14 delivers oxPAPC inside the cell that promotes the inflammasomes-mediated DC and macrophage hyperactivation to release IL-1β without their pyroptosis that increases the inflammation without causing death of the cell and experimental animals subjected to sepsis (282). The CD14 null mice are protected from oxPAPC-mediated inflammation. Further studies are required in this direction in context to the AIM2-ASC inflammasome activation. The deficiency of AIM2-ASC signals forming inflammasomes increases the type 1 IFN production and suppresses protective IFN-γ production (283). In addition to the GSDMD activation, AIM2-ASC-dependent inflammasome formation also inhibits cGAS-STING signaling *via* impeding the STING and TBK1 interaction required for IRF3-dependent type 1 IFN production (283). Hence, AIM2-induced ASC-dependent inflammasome formation has been evolved as an endogenous negative regulator of cGAS-STING signaling-dependent type-1 IFN production to prevent exaggerated inflammation during infections (mycobacterial tuberculosis) and other chronic inflammatory diseases that may cause cancer. Further studies are required in the direction.

### ALRs Recognizing Self-DNAs During Different Inflammatory Diseases or Conditions

ALRs recognize pathogen-derived DNA (bacterial, viral, and parasite-derived) in the cytosol described somewhere else (251, 284). I will discuss here only its role in recognizing self-DNA during different inflammatory conditions or diseases. The AIM2-mediated inflammasome activation has been observed in the influenza virus infection in macrophages due to the release of mtDNA from the infected macrophages (285). The AIM2 recognizes mtDNA that forms ASC-dependent inflammasome and releases mature IL-1β. The mitochondrial ROS production inhibition by Mito-TEMPO (a well-known mitochondria-specific superoxide scavenger) decreases the AIM2-mediated IL-1β production. The AIM2 gene polymorphism is associated with severe periodontitis in patients of northern and western European ancestry with haplotype rs1057028 and rs6940 (a missense SNP) (286). Also, the haplotype with IFI16 (rs6940T-rs855873G) is associated with the increased susceptibility to the Behcet disease (BD), a systemic inflammatory disease involving vasculitis and recurrent mucosal (oral and genital) ulcerations due to the lower expression of IFI16 (287). IFI16-β (a novel transcript isoform of IFI16) is a novel endogenous negative regulator of the AIM2 and blocks the AIM2-ASC complex formation *via* interacting with AIM2 (288). IFI16-β also interacts with the dsDNA and decreases its availability to AIM2, and its enforced expression inhibits AIM2 activation-mediated release of pro-inflammatory cytokines and pyroptosis. The cytosolic IFI16 is functionally similar to mouse p202 (a negative regulator of AIM2).

AIM2 activation also plays a crucial role in dietary steatohepatitis that is further aggravated by the TLR9 activation, which further upregulates AIM2 expression and IL-1β production (289). Along with chronic inflammatory liver conditions, AIM2 activation in Kupffer cells in response to the oxidized mtDNA also plays a crucial role in ischemia-reperfusion-induced hepatitis (290). AIM2 activation also induces joint inflammation in patients with chronic polyarthritis *via* recognizing self-DNA as a DAMP (291). The abdominal aortic aneurysm (AAA) can be an inflammatory AAA (accounts for 5–10% of all aortic aneurysm cases and involves inflammatory immune response localized to the blood vessel wall with unknown mechanism) or a typical atherosclerotic AAA (292, 293). Different inflammatory immune mechanisms play crucial roles in AAA (294, 295). However, a recent study has suggested the activation of AIM2 inflammasomes and dependent pro-inflammatory cytokine release in the mouse model of AAA, and its deficiency has decreased the incidence of AAA in AIM2^-/-^ mice by 48.4% (296). The intravenous injection of poly (deoxyadenylic–deoxythymidylic) acid poly (dA: dT), a synthetic dsDNA releases AIM2 inflammasome activation-dependent pro-inflammatory cytokines (IL-β and IL-18), which dysregulate the reendothelialization of the carotid artery and increase the number of circulating endothelial microparticles (EMP) after acute denudation (297). The subcutaneous poly (dA: dT) injection induces atherosclerotic plaque formation, increases ROS production, and EMP release in the ApoE^-/-^ mice due to AIM2 activation (297). Hence, AIM2 activation plays a crucial role in the atherogenesis and we need further studies in this direction.

AIM2 activation also contributes to the chronic cerebral hypoperfusion-induced brain injury and associated vascular dementia (VaD) *via* promoting apoptotic and pyroptotic cell death pathways (298). AIM2 methylation has also been associated with C-reactive protein (C-RP) polymorphism and C-RP levels in people with post-traumatic stress disorder (PTSD) (299). The inflammasome activation, including AIM2 also contributes to the chronic kidney disease (CKD) and ischemia-reperfusion-induce kidney damage (300, 301). The increase in the mtDNA in the peripheral blood and AIM2 in the monocytes/macrophages of T2DM patients predisposes them to the chronic inflammation and diabetic nephropathy (302–305). Hence, the AIM2 activation plays a crucial role in the inflammatory pathogenesis of many diseases. Thus, further studies are required in the direction to explore the source of AIM2 activation in both animal models and human patients of the disease.

### AIM2 in Cancer

AIM2^-/-^ mice are more susceptible to develop colon cancer following azoxymethane (AOM)-and dextran sulfate sodium (DSS)-induced colitis-associated carcinogenesis due to the uncontrolled proliferation of intestinal stem cells (ISCs) in response to the aberrant activation of Wnt (Wingless and Int-1) signaling and dysbiosis of the gut bacteria (306). However, the protective action of AIM2 against colon cancer is independent of its inflammasome activation mechanism. Also, more than 50% of patients with small bowel cancer have shown a frameshift mutation in the AIM2 gene in patients with hereditary nonpolyposis colorectal cancer (HNPCC) (307). Also, the mismatch repair-deficient colorectal cancers have frequent inactivation of the AIM2 gene (308). Thus mutation inactivating AIM2 functions has been more frequently associated with colon cancer in humans. AIM2 also promotes NSCLC *via* modulating mitochondrial dynamics to promote mitochondrial ROS that promotes MAPK/ERK signaling required for cancer cell growth and proliferation (309, 310). AIM2 also regulates growth and invasion of squamous cell carcinoma (SCC) of the skin or keratinocyte-derived cutaneous squamous cell carcinoma (c-SCC) through increasing the cancer cell viability and invasion as indicated by the increased production of matrix metalloprotease 13 (MMP13) and MMP1 (two proteases with collagen degrading and invasion promoting properties associated with the invasion of c-SCC cells) and vascularization (311).

AIM2 activation also promotes oral squamous cell carcinoma (OSCC) with inactive tumor suppressor p53 *via* increasing cell proliferation and NF-κB activation (312). AIM2 activation in response to the cytosolic dsDNA also plays a significant role in benign prostate hyperplasia (BPH) independent of androgen receptor status (313). However, clinical tumor samples from prostate cancer patients have low mRNA and protein expression of AIM2. Thus, it may be playing a crucial role in the induction of hyperplasia at the initial stages of prostate cancer, and a decrease in its level at later stages may be aggravating the disease that needs further investigation.

AIM2 activation also plays a crucial role in hepatic cancer induced by diethylnitrosamine (DEN)-induced liver damage (314). Kupffer cells of the liver express AIM2 that further increases in response to the DEN-induced liver injury recognizing damage cellular DNAs to produce high levels of pro-inflammatory IL-1β cytokine (314). The genetic deletion of AIM2 has shown a reduction in DEN-induced liver inflammation and hepatic cell carcinoma (HCC) or hepatoma. Hence, AIM2 activation plays a crucial role in HCC. However, another study has shown the loss of AIM2 activity promotes HCC due to the mammalian target of rapamycin (mTOR)-S6K1 (ribosomal protein S6 kinase β1 or p70S6K) pathway promoting proliferation, colony formation, and invasion of HCC cells (315). Hence, AIM2 activation may have a protective role in HCC. Thus these two controversial findings suggest that the cause and stage of cancer may play a crucial role in the AIM2-dependent HCC. More studies are warranted. The mTOR-S6K1 signaling is also pivotal for estrogen receptor (ER) positive breast cancer, and S6K1 serves as a biomarker for prognosis and therapeutic target (316). However, AIM2 activation in breast cancer cell line and the orthotopic mouse model of breast cancer exerts a protective action *via* suppressing NF-κB activation and inducing apoptosis among cancer cells (317). Hence, AIM2 suppression may promote breast cancer pathogenesis as mTOR-S6K1 signaling is increased in patients indicating its activity loss. However, further studies are required to establish this in female breast cancer patients.

## Future Perspectives

The trinity of these cytosolic self-DNA recognizing PRRs plays a crucial role in the maintenance of cellular homeostasis. They serve as guardians of the cellular galaxy for dangers entering the cells, including the pathogens. However, under normal conditions host DNA resides in the nucleus and mitochondria that helps in the development of self-tolerance. TLR9 remains bound to the ER under normal conditions and moves to endosomes as soon as self-DNA moves there from the cytosol. However, whenever cells or tissues undergo stressful conditions, including cellular or mitochondrial one, their genetic material comes into cytosol due to mitochondrial or nuclear damage. Thus physical border preventing their recognition by these PRRs is lost and they become a potential threat to maintaining cellular harmony or homeostasis. Along with, self-DNA other DAMPs, including HMGB1, also come out from the nucleus that further enhances the recognition of self-DNA by TLR9 and cGAS-STING signaling pathways. Looking at their evolutionary origin, cGAS and STING have originated before (approximately 600 MYA) TLRs (around 500 MYA). The recognition of the cytosolic DNA by TLR9 depends on its CpG content, whereas in the case of cGAS, it primarily depends on its length (30–200 bp) and its curvature independent of CpG content. The unmethylated CpG motif content is prevalent in the bacterial DNA, and they are absent in vertebrates generally due to their methylation. Hence, the evolution of TLR9 added to the host defense against pathogens based on CpG that escaped the cGAS recognition. However, the CpG islands in mammals, including humans, may avoid their methylation by directly encoding demethylation signals that are an evolutionarily conserved process (318). Hence, mammalian DNA with CpG in the cytosol also becomes a potential target for TLR9-based recognition and the activation of pro-inflammatory and type 1 IFN signaling. On the other hand, AIM2 also recognizes self-DNA (80–300 bp) independent of CpG content along with pathogen-derived DNA.

ALRs, including AIM2, have evolved in the common ancestors from which marsupials and placental mammals have evolved approximately 200 MYA. Thus it will be interesting to explore the evolutionary forces responsible for AIM2 evolution so late after cGAS and TLR9. As activation of AIM2 *via* GSDMD production inhibits cGAS-STING signaling dependent on type 1 IFN production, one can speculate that it has evolved as a negative regulator of exaggerated inflammation in response to the cGAS activation. For example, cGAS activation in response to the pathogen-derived or self-DNA activates the NLRP3 inflammasome in myeloid cells through inducing lysosomal death that increases the K^+^ efflux, which is one of the crucial factors stimulating NLRP3 inflammasomes and the release of CASP1-dependent pro-inflammatory cytokines (IL-1β and IL-18) (319). The cGAS-induced lysosomal cell death (LCD) in response to the cytosolic DNA involves the trafficking of the activated STING to the lysosome, inducing membrane permeabilization. Another study has indicated the involvement of STING in activating NLRP3 inflammasome formation through recruiting and facilitating NLRP3 localization in the ER during HSV-1 infection (320). STING also attenuates K48 and K63-linked NLRP3 polyubiquitination during viral infection. More studies in this direction will prove helpful to explore the unknown mechanisms regulating the cytosolic PRRs trinity (cGAS, TLR9, and AIM2). However, the location of cGAS (attached to the inner plasma membrane) inside the cell makes it a primary or first response task force among other cytosolic PRRs for invading pathogens or self-DNA of exosomes. For example, phagocytic cells, including macrophages, DCs, and neutrophils have higher distribution of plasma membrane bound cGAS than non-phagocytic cells, indicating its primary role in recognizing invading pathogens. Of note, membrane-bound cGAS has a minimal recognition for self-DNA generated within the cells. On the hand, non-phagocytic cells have larger pools of free cytosolic cGAS as compared to the phagocytic cells. Further studies are crucial in the direction.

These systems have evolved to protect against both outer and internal dangers. However, their overactivation may lead to different inflammatory diseases. Hence, their controlled or regulated function is crucial for maintaining homeostasis. The trinity of these cytosolic PRRs recognizing self-DNA along with pathogen-derived DNA has the potential to serve as potent innate immune system-based immunomodulators and great adjuvants (cGAS and TLR9 activators, CpG ODNs) for better vaccines and optimal immunotherapy for different infectious diseases and cancers (321, 322). For example, streptavidin (secreted by the soil bacteria called *Streptomyces avidinii* with a high affinity for biotin or vitamin B7) activates the cGAS-STING signaling pathway to secrete type 1 IFNs and clears HSV-1 infection that abrogates this signaling pathway-based antiviral immune response (323–325). Streptavidin has been used as an immunostimulator or an adjuvant in cancer vaccines previously without knowing its exact mode of action on innate immunity (326). Hence, streptavidin can be used with caution with specificity through its stimulatory action on cGAS-STING signaling-mediated type 1 IFN production. The cGAMP-mediated STING activation has been found effective for cutaneous vaccination as a potent adjuvant without undesired skin irritation (327).

The antitumor effect of the antidiabetic drug metformin occurs through activating the STING/IRF3/IFN-β pathway *via* inhibiting AKT signaling in pancreatic cancer cells, including pancreatic ductal adenocarcinoma (PDAC) (328). Hence, the recognition of this cytosolic trinity of PRRs detecting cytosolic DNA has explained the previously unknown mechanisms of drugs used in clinics, which can be used in the future for other diseases depending on the involvement of these PRRs in the diseases. Also, the STING-based biosensor called BioSTING has been developed to detect CDNs in eukaryotic cells that will prove beneficial in diagnosing different cancers and other inflammatory diseases, including autoimmune or autoinflammatory ones (329). However, caution should be taken to use cGAS and TLR9-based adjuvants as their overactivation is associated with different autoinflammatory or autoimmune diseases (ADs) and other sterile inflammatory conditions. Hence, the homeostasis of the cellular galaxy is maintained till the sleeping status of the trinity of cytosolic PRRs is maintained, their aggressive awakening in response to indigenous DAMPs proves lethal to the cell causing its death (apoptosis, lysosomal cell death, pyroptosis, and necroptosis) and induces different immune-mediated inflammatory diseases.

## Author Contributions

The author has developed the idea, designed, and wrote the manuscript, along with designing and developing the figures.

## Conflict of Interest

The author declares that the research was conducted in the absence of any commercial or financial relationships that could be construed as a potential conflict of interest.

## Abbreviations

AA, amino acid; AAA, abdominal aortic aneurysm; ADs, autoimmune or autoinflammatory diseases; AGO2, argonaute RISC catalytic component 2; AGS, Aicardi-Goutières syndrome; aHSCT, allogenic hematopoietic stem cell transplantation; AIM2, absent in melanoma 2; ALRs, AIM-2-like receptors; ALD, alcohol-related liver disease; AMP, adenosine monophosphate; ARF, ADP ribosylation factor; ALS, amyotrophic lateral sclerosis; ApoE, apolipoprotein E; Arp2/3 complex, actin-related proteins-2/3 complex; ATG5, autophagy-related protein 5; Atg9a, autophagy-related protein 9a; ATG16L1, autophagy-related protein 16 like 1; BAF, Barrier-to-autointegration factor 1; BCR, B cell receptor; BD, Behcet disease; BECN1, Beclin1; bp, base pairs; BPD, bronchopulmonary dysplasia; BS, Bloom syndrome; Btk, Bruton’s tyrosine kinase; CARD15, caspase recruitment domain-containing protein 15; CASP1, Caspase 1; cGAS, cyclic GMP-AMP synthase; CDK, Cyclin-dependent kinase; c9orf72, chromosome 9 open reading frame 72; CLP, Cecal-ligaiton and puncture; CLRs, C-type lectin-like receptors; COPI or II, Coat protein complex-I or II; COPA, Coatomer protein subunit alpha; CREB, cyclic response element-binding protein; CTT, C-terminal tail; DDR, DNA damage response; DDX41, DEAD (Asp-Glu-Ala-Asp) box polypeptide 41; DNA-PK, DNA-dependent protein kinase; DNMT1, DNA (cytosine-5)-methyltransferase 1; EAE, experimental autoimmune encephalitis; FCVs, E cigarette vapours; EIA, erosive inflammatory arthritis; ERGIC, endoplasmic reticulum Golgi-intermediate compartment; ESCRT, endosomal sorting complex required for transport; EV71, enterovirus 71; FTD, Frontotemporal dementia; GMP, Guanosine monophosphate; GSDMD, Gasdermin D; GVHD, Graft-versus-host disease; HDAC3, Histone deacetylase 3; HFMD, head, foot, and mouth disease; HIN, hematopoietic expression, interferon-inducible nature, and nuclear localization; HMGB1, high mobility group box protein 1; HNPCC, hereditary nonpolyposis colorectal cancer; HOMA-IR, homeostasis model assessment of insulin resistance; Iba1, ionized calcium-binding adapter molecule 1; ICAM-1, intercellular adhesion molecule-1; IFI16, gamma-interferon-inducible protein Ifi-16; IKKβ, inhibitor of nuclear factor kappa-B kinase subunit β; Imd, immune cell deficiency; IRAK1, interleukin-1 receptor-associated kinase-1; IRAK-4, interleukin-1 receptor-associated kinase-4; IRE1α, Inositol-requiring transmembrane kinase/endoribonuclease 1α; IRF3, Interferon regulatory factor 3; IRGM, immunity-related GTPase M; LC3, microtubule-associated protein 1 light chain 3; LSM11, U7 small nuclear RNA associated protein; LysRS, Lysyl-tRNA synthetase; MALAT1, metastasis-associated lung adenocarcinoma transcript 1; MAVS, mitochondrial antiviral signaling protein; MB21D1, MAB-21 domain containing protein 1; MCP1, monocyte chemotactic protein 1; MNDA, myeloid cell nuclear differentiation antigen; MS, multiple sclerosis; MST1, mammalian Sterile 20-like kinases 1; MYA, million years ago; MYSM1, Myb-like, SWIRM, and MPN domains 1 protein; NAFLD, non-alcoholic fatty liver disease; NASH, non-alcoholic steatohepatitis; NEAT1, nuclear paraspeckle assembly transcript 1; NEMO, NF-κB essential modulator, NET, neutrophil extracellular trap; NLRs, NOD-like receptors; NOD, nucleotide-binding oligomerization domain; NONO, non-POU domain containing octamer binding; NSCLC, non-small cell lung cancer; nts, nucleotides or nucleotide sequence; OCT, optical coherence tomography; ODNs, oligodeoxyribonucleotides; PA, palmitic acid; PDL-1, programmed death ligand 1; PI3K, phosphoinositide 3-kinase; PINK1, phosphatase and tensin homolog-induced kinase 1; PIP2; Phosphatidylinositol 4,5 biphosphate or PtdIns(4,5)P2; PLD3 and 4, phospholipase D3/4; PRKDC, Protein Kinase, DNA-activated, catalytic subunit; PYCARD, PYD and CARD containing protein; PYHIN1, pyrin and HIN domain-containing protein 1; RIG-1, retinoic acid inducible gene-1; RISC, RNA-induced silencing complex; RLRs, RIG-1-like receptors; RIP2, receptor interacting protein 2; RNU7-1, U7 small nuclear 1; SASP, senescence-associated secretory phenotype; SAVI, STING-associated vasculopathy with onset in infancy; SCC, squamous cell carcinoma; SHIP-1, SH-2 containing inositol 5’ polyphosphatase 1; SREBP2, sterol regulatory element-binding protein 2; STING, stimulating interferon genes; STK11, serine-threonine kinase 11; TAB 1, TAK1 binding protein 1; TAB2, TAK1 binding protein 2; TAK1, yransforming growth factor-β associated kinase 1; TANK, TRAF-associated NF-κB activator; TBK1, TANK-binding kinase 1; TFAM, mitochondrial transcription factor A; TMEM203, transmembrane protein 203; TMS1, target of methylation-induced silencing-1; TOLLIP, Toll-interacting protein; TRAF6, TNFR-associated factor 6; TREX1, three prime repair exonuclease 1; ULK, Unc-51-like kinase; UNC93B1, Unc-93 homolog B1; UPR, unfolded protein response; VPS34, vacuolar protein sorting 34; WASP, Wiskott-Aldrich syndrome protein; WD40 CTD, WD repeat-containing C-terminal domain; WIPI2, WD repeat domain phosphoinositide-interacting *protein* 2; WT, Wild type; YAP, Hippo-Yes-associated protein.

## Glossary

**Table:**

AA	Amino acid
AAA	Abdominal aortic aneurysm
Ads	Autoimmune or autoinflammatory diseases
AGO2	Argonaute RISC Catalytic Component 2
AGS	Aicardi-Goutières syndrome
aHSCT	allogenic hematopoietic stem cell transplantation
AIM2	Absent in melanoma 2
ALRs	AIM-2-like receptors
ALD	Alcohol-related liver disease
AMP	Adenosine monophosphate
ARF	ADP ribosylation factor
ALS	Amyotrophic lateral sclerosis
ApoE	Apolipoprotein E
**Arp2/3 complex**	Actin-related proteins-2/3 **complex**
ATG5	Autophagy-related protein 5
Atg9a	Autophagy-related protein 9a
ATG16L1	Autophagy-related protein 16 like 1
BAF	Barrier-to-autointegration factor 1
BCR	B cell receptor
BD	Behcet disease
BECN1	Beclin1
bp	Base pairs
BPD	Bronchopulmonary dysplasia
BS	Bloom syndrome
Btk	Bruton’s tyrosine kinase
CARD15	Caspase recruitment domain-containing protein 15
CASP1	Caspase 1
cGAS	cyclic GMP-AMP synthase
CDK	Cyclin-dependent kinase
c9orf72	chromosome 9 open reading frame 72
CLP	Cecal-ligaiton and puncture
CLRs	C-type lectin-like receptors
COPI or II	Coat protein complex-I or II
COPA	Coatomer protein subunit alpha
CREB	cyclic response element-binding protein
CTT	C-terminal tail
DDR	DNA damage response
DDX41	DEAD (Asp-Glu-Ala-Asp) box polypeptide 41
DNA-PK	DNA-dependent protein kinase
DNMT1	DNA (cytosine-5)-methyltransferase 1
EAE	Experimental autoimmune encephalitis
FCVs	E cigarette vapours
EIA	Erosive inflammatory arthritis
ERGIC	Endoplasmic reticulum Golgi-intermediate compartment
ESCRT	Endosomal sorting complex required for transport
EV71	Enterovirus 71
FTD	Frontotemporal dementia
GMP	Guanosine monophosphate
GSDMD	Gasdermin D
GVHD	Graft-versus-host disease
HDAC3	Histone deacetylase 3
HFMD	Head, foot, and mouth disease
HIN	Hematopoietic expression interferon-inducible nature, and nuclear localization
HMGB1	High mobility group box protein 1
HNPCC	Hereditary nonpolyposis colorectal cancer
HOMA-IR	Homeostasis model assessment of insulin resistance
Iba1	Ionized calcium-binding adapter molecule 1
ICAM-1	Intercellular adhesion molecule-1
IFI16	Gamma-interferon-inducible protein Ifi-16
IKKβ	Inhibitor of nuclear factor kappa-B kinase subunit β
Imd	Immune cell deficiency
IRAK1	Interleukin-1 receptor-associated kinase-1
IRAK-4	Interleukin-1 receptor-associated kinase-4
IRE1α	Inositol-requiring transmembrane kinase/endoribonuclease 1α
IRF3	Interferon regulatory factor 3
IRGM	Immunity-related GTPase M
LC3	Microtubule-associated protein 1 light chain 3
LSM11	U7 small nuclear RNA associated protein
LysRS	Lysyl-tRNA synthetase
MALAT1	Metastasis-associated lung adenocarcinoma transcript 1
MAVS	Mitochondrial antiviral signaling protein
MB21D1	MAB-21 domain containing protein 1
MCP1	Monocyte chemotactic protein 1
MNDA	Myeloid cell nuclear differentiation antigen
MS	Multiple Sclerosis
MST1	Mammalian Sterile 20-like kinases 1
MYA	Million years ago
MYSM1	Myb-like, SWIRM, and MPN domains 1 protein
NAFLD	Non-alcoholic fatty liver disease
NASH	Non-alcoholic steatohepatitis
NEAT1	Nuclear paraspeckle assembly transcript 1
NEMO	NF-κB essential modulator
NET	Neutrophil extracellular trap
NLRs	NOD-like receptors
NOD	Nucleotide-binding oligomerization domain
NONO	Non-POU Domain Containing Octamer Binding
NSCLC	Non-small cell lung cancer
nts	nucleotides or nucleotide sequence
OCT	Optical coherence tomography
ODNs	Oligodeoxyribonucleotides
PA	Palmitic acid
PDL-1	Programmed death ligand 1
PI3K	Phosphoinositide 3-kinase
PINK1	Phosphatase and tensin homolog-induced kinase 1
PIP2	Phosphatidylinositol 4,5 biphosphate or PtdIns(4,5)P2
PLD3 and 4	Phospholipase D3/4
PRKDC	Protein Kinase
DNA-Activated	Catalytic Subunit
PYCARD	PYD and CARD containing protein
PYHIN1	Pyrin and HIN domain-containing protein 1
RIG-1	Retinoic acid inducible gene-1
RISC	RNA-induced silencing complex
RLRs	RIG-1-like receptors
RIP2	Receptor interacting protein 2
RNU7-1	U7 small nuclear 1
SASP	Senescence-associated secretory phenotype
SAVI	STING-associated vasculopathy with onset in infancy
SCC	Squamous cell carcinoma
SHIP-1	SH-2 containing inositol 5' polyphosphatase 1
SREBP2	sterol regulatory element-binding protein 2
STING	Stimulating interferon genes
STK11	Serine-threonine kinase 11
TAB 1	TAK1 binding protein 1
TAB2	TAK1 binding protein 2
TAK1	Transforming growth factor-β associated kinase 1
TANK	TRAF-associated NF-κB activator
TBK1	TANK-binding kinase 1
TFAM	Mitochondrial transcription factor A
TMEM203	Transmembrane protein 203
TMS1	Target of Methylation-induced Silencing-1
TOLLIP	Toll-interacting protein
TRAF6	TNFR-associated factor 6
TREX1	Three prime repair exonuclease 1
ULK	Unc-51-like kinase
UNC93B1	Unc-93 Homolog B1
UPR	Unfolded protein response
VPS34	vacuolar protein sorting 34
WASP	Wiskott-Aldrich syndrome protein
WD40 CTD	WD repeat-containing C-terminal domain
WIPI2	WD repeat domain phosphoinositide-interacting *protein* 2
WT	Wild type
YAP	Hippo-Yes-associated protein
